# Genomic imprinting analyses identify maternal effects as a cause of phenotypic variability in type 1 diabetes and rheumatoid arthritis

**DOI:** 10.1038/s41598-020-68212-x

**Published:** 2020-07-14

**Authors:** Inga Blunk, Hauke Thomsen, Norbert Reinsch, Manfred Mayer, Asta Försti, Jan Sundquist, Kristina Sundquist, Kari Hemminki

**Affiliations:** 1 Institute of Genetics and Biometry, Leibniz Institute for Farm Animal Biology (FBN), Wilhelm-Stahl-Allee 2, 18196 Dummerstorf, Germany; 2 Division of Molecular Genetic Epidemiology, German Cancer Research Centre (DKFZ), Heidelberg, Germany; 3 GeneWerk GmbH, Heidelberg, Germany; 4 Center for Primary Health Care Research, Lund University, Malmö, Sweden; 5 Hopp Children’s Cancer Center (KiTZ), Heidelberg, Germany; 6 Division of Pediatric Neurooncology, German Cancer Research Center (DKFZ), German Cancer Consortium (DKTK), Heidelberg, Germany; 7 Department of Family Medicine and Community Health, Department of Population Health Science and Policy, Icahn School of Medicine at Mount Sinai, New York, USA; 8 Center for Community-Based Healthcare Research and Education (CoHRE), Department of Functional Pathology, School of Medicine, Shimane University, Izumo, Japan; 9 Faculty of Medicine and Biomedical Center in Pilsen, Charles University in Prague, Pilsen, Czech Republic

**Keywords:** Population genetics, Genetics, Epigenetics, Imprinting

## Abstract

Imprinted genes, giving rise to parent-of-origin effects (POEs), have been hypothesised to affect type 1 diabetes (T1D) and rheumatoid arthritis (RA). However, maternal effects may also play a role. By using a mixed model that is able to simultaneously consider all kinds of POEs, the importance of POEs for the development of T1D and RA was investigated in a variance components analysis. The analysis was based on Swedish population-scale pedigree data. With *P* = 0.18 (T1D) and *P* = 0.26 (RA) imprinting variances were not significant. Explaining up to 19.00% (± 2.00%) and 15.00% (± 6.00%) of the phenotypic variance, the maternal environmental variance was significant for T1D (*P* = 1.60 × 10^−24^) and for RA (*P* = 0.02). For the first time, the existence of maternal genetic effects on RA was indicated, contributing up to 16.00% (± 3.00%) of the total variance. Environmental factors such as the social economic index, the number of offspring, birth year as well as their interactions with sex showed large effects.

## Introduction

The failure of the immune system to distinguish self from non-self antigens is the basis for autoimmune disorders (AIs)^1^. Type I diabetes (T1D) is an AI that causes chronic destruction of pancreatic islet ß-cells and hyperglycemia due to reduced insulin production^2^. With the incidence said to be increasing by 3–4% yearly, more than 20 million individuals are estimated to have T1D worldwide^3^. Rheumatoid arthritis (RA) is associated with autoantigen presentation with antigen specific T and B cell activation and aberrant inflammatory cytokine production. Consequences thereof include synovitis, proliferation of synovia and cartilage, and subchondral bone destruction^4^. The occurrence of RA is relatively constant and ranges between 0.5 and 1.0% in European and North-American populations^5^. The exact etiology of T1D and RA remains largely unknown^1^, however, a complex interplay of genetic, environmental, and epigenetic factors is assumed^4,6,7^.

With regard to genetic factors, the strongest effects have been found within the major histocompatibility complex or human leukocyte antigen system. T1D and RA show genetic overlap in terms of associations within *HLA*, *PTPN22*, *CTLA4*, and *TAGAP*^8^. Causal loci explain over 80% of T1D heritability which reportedly ranges between 40 and 92%^3^. For RA, associated variants outside and inside of the major histocompatibility complex region explain about 5% and 60% of the heritability^9,10^. Heritability estimates range between 12 and 60%^11–13^.

As disorder concordance rates in monozygotic twins have been observed to be less than 100%, AIs are assumed to be subject to epigenetic modifications^4,14–16^. Perhaps the best-known example for all epigenetic phenomena is imprinting, in which the expression of genes is either maternally or paternally inactivated. Inactivation can either be full or partial^17^. Partial imprinting occurs when the inactivation of alleles is not complete. For example, loci may be imprinted in a tissue-specific manner^18^, or the imprinting status varies over time during the developmental stages^19^. As they appear as phenotypic differences between heterozygotes depending on their parental allele origin, imprinting effects belong to the class of parent-of-origin effects (POEs)^20^. Imprinting has been identified in mammals, insects, and plants^21^. It is nevertheless assumed that less than 1% of all genes in mammals are imprinted^20,22^, however, they have important functions in stem cells, neuronal differentiation, development, and growth^22,23^. In humans, imprinted genes are associated with diseases such as Prader–Willi syndrome^24^, Angelman syndrome^25^, and cancer^26,27^. They are also assumed to affect susceptibility to diabetes. This assumption originates from observations that T1D is preferentially expressed by children of T1D-affected fathers^28–30^. Whether this observation is due to imprinting or other factors is not clear since findings are contradictory^29,31^. With regard to RA, the existence of imprinting has been discussed since its incidence is considerably higher in women than in men^32^. However, imprinting studies are rare and results have been inconclusive; the role of imprinting in RA susceptibility is therefore not yet understood^33,34^. Imprinted genes are difficult to detect in conventional association studies as their effects depend on the parental origin of the risk allele^35^. The incorporation of knowledge on whether imprinting affects susceptibility to T1D and RA could increase the power to find causal genes^34^. Moreover, the development of therapeutic approaches targeting these genes or their regulators could be improved^33^. Therefore, the first goal of this study was to investigate the impact of imprinting on the susceptibility to T1D and RA in a variance components analysis by applying a unique mixed model (*imprinting model*). The model allows for the simultaneous consideration of all kinds of imprinting patterns (full, partial, maternal, and paternal). As it has never been applied to human population data before, it opens up new opportunities for understanding the etiology of T1D and RA^17,36–40^.

In an imprinting variance components analysis, maternal effects must be accounted for in the model to avoid inflated estimates^41^. The second research goal was therefore to incorporate maternal effects into the statistical model; not only to prevent biases in the imprinting variances, but also to investigate the maternal contribution to T1D and RA susceptibility. Like imprinting effects, maternal effects contribute to the broader class of POEs. However, their variation is assigned to the environmental contribution to the phenotypic variance. According to Falconer^42^, they are defined as prenatal and postnatal effects on offspring and can have two main components. The first component is the maternal genotypic effect on, for example, the birthweight of her children (maternal genetic effect)^43^. The second component is the maternal environmental effect on the birthweight of her offspring^42^. This component refers to the permanent environmental effects of the mother on all of her offspring and can therefore also be considered a shared household effect^42^. Although T1D and RA differ in their average age of onset, attention must be given to the maternal contribution in the development of both diseases since early environmental factors can permanently modify the development of the immune system^44^. The *imprinting model* is able to separate maternal effects from maternal imprinting effects, allowing the first imprinting variance components analysis to be performed in human population genetics.

The third goal of this study was to gain knowledge on the importance of sex and environmental triggers such as birth year, social economic index, and the number of offspring on the susceptibility to T1D and RA. Overall, this study brought to light the complex interplay between genetic, epigenetic and environmental factors in the development of autoimmunity.

## Theory

A unique mixed model, previously used on animal data, was applied to investigate the existence of imprinting^36–40,45^. The advantage this model confers is that it is able to simultaneously consider all kinds of imprinting (i.e. maternal, paternal, full, and partial imprinting) in its analyses^17^, ultimately separating maternal imprinting effects from maternal “non-imprinting” effects (e.g., maternal environmental and maternal genetic effects). This was not possible with previous population-scale imprinting analyses models, for example, that of Engellandt and Tier^46^. Our *imprinting model* estimates two parental gametic variances and one covariance simultaneously. It is written as:

$${\varvec{Y}} = {\varvec{Xb}} + {\varvec{Z}}_{{\varvec{s}}} {\varvec{g}}_{{\varvec{s}}} + {\varvec{Z}}_{{\varvec{d}}} {\varvec{g}}_{{\varvec{d}}} + {\varvec{e}},\quad \quad \quad \quad \quad \quad (imprinting\;model)$$

where ***Y*** is a vector of the response variable; ***b*** is a vector of fixed effects; ***g***_***s***_ is the vector of random gametic effects under a paternal expression pattern; ***g***_***d***_ is the vector of random gametic effects under a maternal expression pattern; ***X***, ***Z***_***s***_, and ***Z***_***d***_ are the corresponding incidence matrices; and ***e*** is the vector of random residuals. The variance–covariance structure is:

$${\text{Var}}\left[ {\begin{array}{*{20}c} {{\varvec{g}}_{{\varvec{s}}} } \\ {{\varvec{g}}_{{\varvec{d}}} } \\ \end{array} } \right] = G \otimes \left[ {\begin{array}{*{20}c} {\sigma_{s}^{2} } & {\sigma_{sd} } \\ {\sigma_{sd} } & {\sigma_{d}^{2} } \\ \end{array} } \right],$$

where $\sigma_{s}^{2}$ and $\sigma_{d}^{2}$ are the gametic variances and $\sigma_{sd}$ is the covariance. Matrix ***G*** is the gametic relationship matrix reflecting the relationships between the gametes of all individuals in a pedigree. It is therefore twice the size of the number of individuals included in the analysis^47,48^. The symbol ⊗ denotes the Kronecker product. The imprinting effect is defined as the vector of differences (***g***_***s***_ − ***g***_***d***_) and the corresponding variance of differences is $\sigma_{i}^{2} = \sigma_{s}^{2} + \sigma_{d}^{2} {-} 2\sigma_{sd}$, which represents the imprinting variance. Where no imprinting is observed, $\sigma_{s}^{2} = \sigma_{d}^{2} = \sigma_{sd}$ and $\sigma_{i}^{2} = 0$.

## Results

### Parent-of-origin effects

#### Type 1 diabetes

##### Genomic imprinting

Using a REML log-likelihood ratio test (RLRT), the significance of the imprinting variance was tested by comparing the logarithmic value of the restricted maximum likelihood (REML log-likelihood) of the linear *imprinting model* to the REML log-likelihood outcome of a corresponding linear *Mendelian model* (equivalent null model that assumes the non-existence of imprinting). At a 5% significance level, the analysis revealed that imprinted genes did not significantly contribute to the total genetic variance in T1D susceptibility in the Swedish population data (*P* = 0.18).

##### Maternal effects

**Figure 1:**
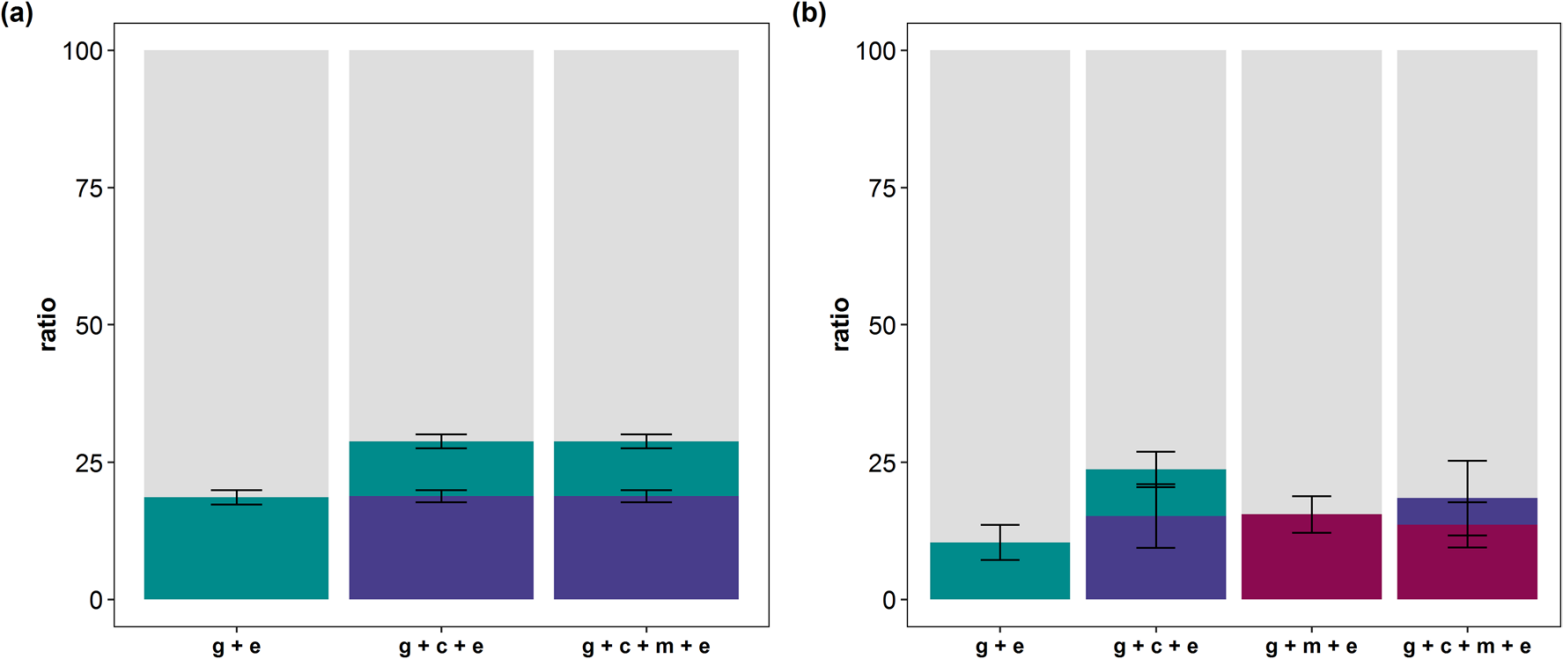
Phenotypic variance of type 1 diabetes (**a**) and rheumatoid arthritis (**b**) is partitioned into the residual variance (gray), additive genetic variance (green), maternal environmental variance (blue), and maternal genetic variance (red). The variance components were estimated using models with a gametic effect (*g*), a maternal environmental effect (*c*), a maternal genetic effect (*m*), and a residual (*e*). Standard errors are indicated by error bars.

Initially, data were analysed using linear models in order to test the significance of the variance components. First, a linear model that ignored maternal effects was applied, i.e. only the genetic effect of the individual was included in the model (*Mendelian model 1*). This led to a T1D heritability estimate (*h*^2^) of 0.19 (± 0.1 × 10^−1^), i.e. 19% of the phenotypic variation in T1D is due to the variation in genetic factors in the analysed population (Fig. 1). Adding a maternal environmental effect to the model (*Mendelian model 2*) revealed significant maternal environmental variance with *P* = 1.60 × 10^−24^. The relative maternal T1D environmental variance was 0.19 (± 0.2 × 10^−1^), i.e. 19% of the phenotypic variance in T1D is due to the variation in maternal environmental effects (Fig. 1). Heritability was reduced to 0.10 (± 0.1 × 10^−1^). Augmentation of *Mendelian model 2* by the maternal genetic effect (*Mendelian model 3*) did not change the REML log-likelihood or variance component ratios (Fig. 1). More detailed information on the variance component estimates in T1D and REML log-likelihood models is provided in Supplementary Table S1.

In addition to the linear models, threshold models were applied to account for the binary nature of the phenotypic traits. However, each of the threshold models could only pick up one variance component, i.e. with the addition of parameters, the same amount of variation was explained by additive genetic effects, then by maternal environmental effects, and then by maternal genetic effects (Table 1).

**Table 1:** Heritability (*h*^2^), relative maternal environmental variance (*c*^2^), and relative maternal genetic variance (*m*^2^) for type 1 diabetes (T1D) and rheumatoid arthritis (RA) estimated using threshold models with a gametic effect (*g*), a maternal environmental effect (*c*), a maternal genetic effect (*m*), and a residual effect (*e*).

	*Mendelian model 1*		*Mendelian model 2*		*Mendelian model 3*	
	*g* + *e*		*g* + *c* + *e*		*g* + *c* + *m* + *e*	
**T1D**						
*h*^2^	0.44 × 10^−1^	(± 0.1 × 10^−1^)	0.00	(± 0.0)	0.00	(± 0.0)
*c*^2^			0.47 × 10^−1^	(± 0.1 × 10^−1^)	0.00	(± 0.0)
*m*^2^					0.52 × 10^−1^	(± 0.1 × 10^−1^)
**RA**						
*h*^2^	0.26 × 10^−1^	(± 0.2 × 10^−1^)	0.26 × 10^−1^	(± 0.2 × 10^−1^)	0.65 × 10^−2^	(± 0.1)
*c*^2^			0.00	(± 0.0)	0.00	(± 0.0)
*m*^2^					0.20 × 10^−1^	(± 0.1)

Standard errors are in parentheses.

#### Rheumatoid arthritis

##### Genomic imprinting

As maternally derived environmental and genetic effects could not be unambiguously disentangled, the *imprinting model* was applied in two forms: (a) with only the maternal environmental effect in addition to the two parental gametic effects, and (b) with only the maternal genetic effect in addition to the two parental gametic effects. The RLRT of model version (a) did not indicate significant imprinting variance (*P* = 0.26). Model version (b) led to a REML log-likelihood of 8,408.70, which was slightly smaller than the REML log-likelihood obtained from the corresponding null model containing a gametic effect and a maternal genetic effect (8,408.88). Because the addition of a parameter to a model should result in an REML log-likelihood value either being equal to or larger than that found here, these results could indicate a flat likelihood surface or numerical inaccuracies.

##### Maternal effects

Maternal effects were initially ignored (*Mendelian model 1*), which resulted in an *h*^2^ value of 0.10 (± 0.3 × 10^−1^; Fig. 1). Following the inclusion of a maternal environmental effect (*Mendelian model 2*), the *h*^2^ value was reduced to 0.85 × 10^−1^ (± 0.3 × 10^−1^). The corresponding maternal variance component was significant at a 5% significance level (*P* = 0.02). The relative maternal environmental variance was 0.15 (± 0.6 × 10^−1^; Fig. 1). While the REML log-likelihood value was not significantly altered upon addition of the maternal genetic effect (*P* = 0.21; *Mendelian model 3*), the relative maternal RA genetic variance estimate was 0.14 (± 0.4 × 10^−1^; Fig. 1), the *h*^2^ estimate dropped to zero and the relative maternal environmental variance was reduced from 0.15 (± 0.6 × 10^−1^) to 0.49 × 10^−1^ (± 0.7 × 10^−1^). To investigate the importance of maternal genetic effects in more detail, a linear model that corresponded to the *Mendelian model 2* but substituted the maternal environmental effect with maternal genetic effect was applied. The application of this model resulted in a relative maternal genetic variance of 0.16 (± 0.3 × 10^−1^) and an *h*^2^ estimate of zero (Fig. 1). The RLRT indicated a significantly better fit in comparison to *Mendelian model 1* (*P* = 0.01). Comparing the results to those associated with *Mendelian model 3*, the REML log-likelihood was not significantly different (*P* = 0.53). Detailed information on RA variance component estimates and REML log-likelihoods of the models is provided in Supplementary Table S2.

The threshold version of *Mendelian model 1* resulted in an *h*^2^ of 0.26 × 10^−1^ (± 0.2 × 10^−1^). As an equal additive genetic variance, and thus the same *h*^2^, was found using the threshold version of *Mendelian model 2*, maternally derived environmental factors appeared not to play a role (Table 1) in RA. However, when the maternal genetic effect was added (*Mendelian model 3*) the *h*^2^ value was 0.65 × 10^−2^ (± 0.1), while the maternal environmental variance remained zero and the relative maternal genetic variance was 0.20 × 10^−1^ (± 0.1; Table 1).

### Environmental and sex effects

#### Type 1 diabetes

##### Birth year

**Figure 2:**
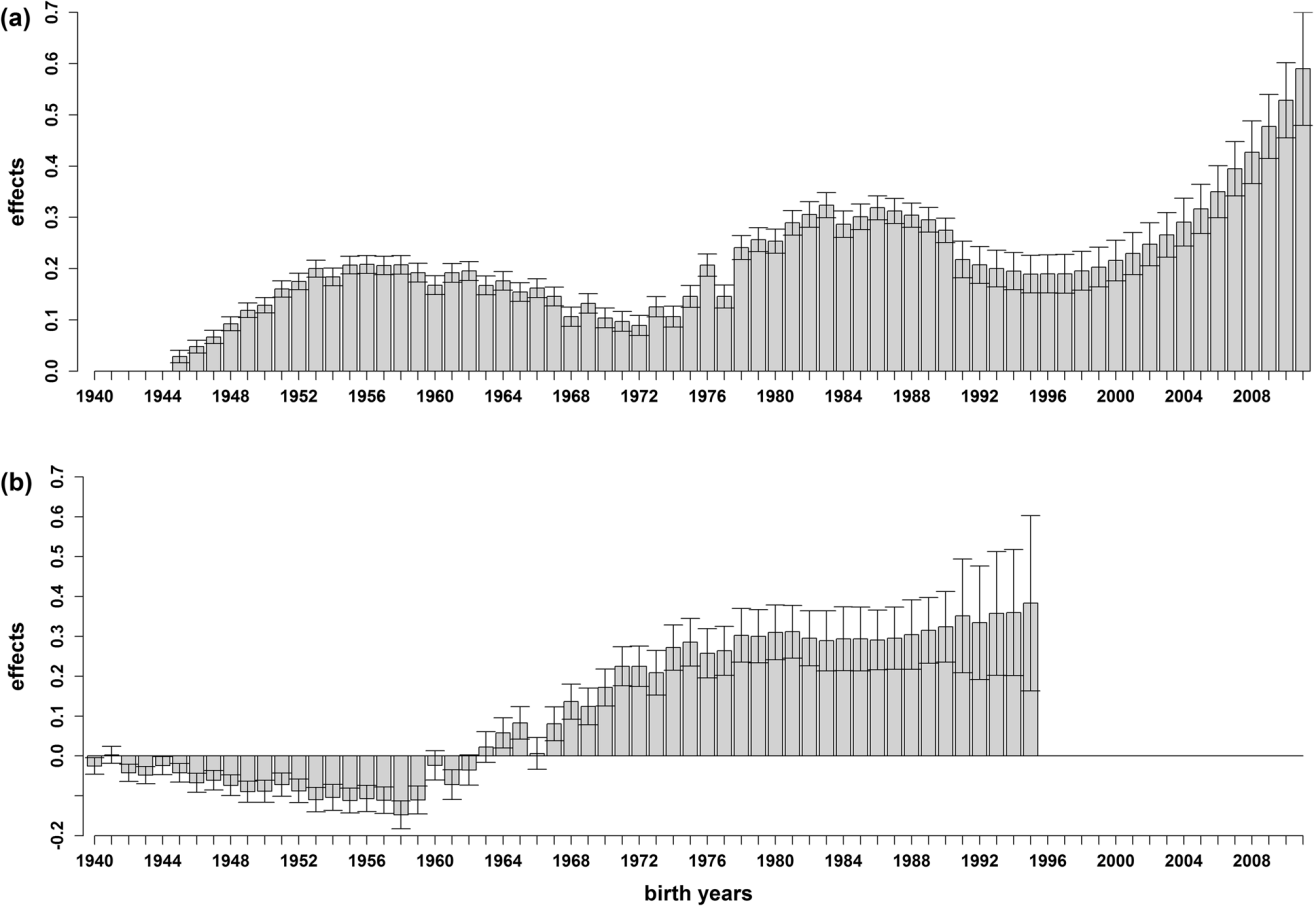
Effects of birth years on the susceptibility to type 1 diabetes (**a**) and rheumatoid arthritis (**b**). For type 1 diabetes, effects were estimated using a linear mixed model that includes a random gametic effect and a random maternal environmental effect. For rheumatoid arthritis a random maternal genetic effect was added. The standard errors are indicated by error bars.

Across all models (including linear and threshold models), the effects of the year of birth (ranging from 1944 to 2012) were shown to differ significantly (*P* < 1.00 × 10^−3^; Table 2; Supplementary Table S3). Effects increased until the end of the 1950s and started declining slightly at the beginning of the 1960s. The effects increased after 1972 until the mid-1980s, declined again until the mid-1990s, and have been increasing ever since (Fig. 2). With the exception of a strong increase of effects and standard errors in 1992 when applying the threshold models (data not shown), trends observed and effects generated under the threshold models were in accordance with those observed for the linear models.

**Table 2:** Overview of incremental Wald *F* values (*F*), number of numerator degrees of freedom (*DF*), number of denominator degrees of freedom (*DF*_*den*_), and the *P* values (*P*) for all fixed effects on type 1 diabetes (T1D) and rheumatoid arthritis (RA), which were sex, birth year, social economic index (SEI), number of offspring (no. offspring), medical region, SEI of the mother (SEI_mother_), years under observation (years_obs_), and whether an individual was a single child or not (single child).

Effect	*DF*	*Mendelian model 1*			*Mendelian model 2*			*Mendelian model 3*			*imprinting model*		
		*g* + *e*			*g* + *c* + *e*			*g* + *c* + *m* + *e*			*g*_*s*_ + *g*_*d*_ + *c* + *e*		
		*DF*_*den*_	*F*	*P*	*DF*_*den*_	*F*	*P*	*DF*_*den*_	*F*	*P*	*DF*_*den*_	*F*	*P*
**T1D**													
Birth year	67	68,461.1	1,696.76	0.00	69,638.8	1,579.83	0.00	69,631.0	1,579.70	0.00	69,586.8	1,577.98	0.00
SEI_mother_	5	70,870.5	12.35	5.35 × 10^−12^	68,490.8	12.64	2.68 × 10^−12^	68,489.6	12.64	2.68 × 10^−12^	68,395.0	12.66	2.56 × 10^−12^
Sex	1	70,917.7	1,032.05	8.05 × 10^−225^	70,779.3	971.73	6.88 × 10^−212^	70,779.3	971.68	7.05 × 10^−212^	70,464.8	892.65	6.45 × 10^−195^
Sex*birth year	67	69,252.5	6.12	1.50 × 10^−50^	70,163.3	6.12	1.50 × 10^−50^	70,157.3	6.12	1.50 × 10^−50^	70,105.4	6.11	1.98 × 10^−50^
Medical region	25	50,691.5	32.97	2.12 × 10^−156^	47,088.9	32.50	7.34 × 10^−154^	47,120.5	32.50	7.33 × 10^−154^	47,038.1	32.52	5.80 × 10^−154^
Years_obs_	3	70,670.0	1,086.26	0.00	70,689.1	1,086.68	0.00	70,687.3	1,086.68	0.00	70,674.5	1,086.92	0.00
**RA**													
Birth year	56	20,417.2	26.69	3.34 × 10^−265^	20,513.0	25.01	7.06 × 10^−247^	20,356.1	24.54	1.14 × 10^−241^	20,067.2	23.07	1.84 × 10^−225^
SEI	5	20,858.0	9.89	1.84 × 10^−9^	20,853.7	9.93	1.68 × 10^−9^	20,851.0	9.94	1.64 × 10^−9^	20,811.1	9.98	1.49 × 10^−9^
SEI_mother_	5	20,845.5	2.98	0.11 × 10^−1^	20,194.7	2.99	0.11 × 10^−1^	20,068.6	2.95	0.12 × 10^−1^	20,134.7	2.98	0.11 × 10^−1^
Sex	1	20,852.5	686.42	6.86 × 10^−149^	20,829.9	680.46	1.24 × 10^−147^	20,856.2	642.81	1.06 × 10^−139^	20,850.8	620.47	5.54 × 10^−135^
Sex*birth year	56	20,516.0	1.38	0.32 × 10^−1^	20,607.1	1.38	0.32 × 10^−1^	20,526.9	1.38	0.32 × 10^−1^	20,170.0	1.39	0.29 × 10^−1^
Sex*SEI	5	20,833.9	6.29	7.71 × 10^−6^	20,798.3	6.24	8.64 × 10^−6^	20,815.4	6.26	8.25 × 10^−6^	20,729.7	6.26	8.25 × 10^−6^
Sex*no. offspring	11	20,799.0	8.58	2.51 × 10^−15^	20,605.9	8.59	2.39 × 10^−15^	20,587.5	8.57	2.64 × 10^−15^	20,256.6	8.58	2.52 × 10^−15^
No. offspring	11	20,681.9	175.65	0.00	20,472.8	175.61	0.00	20,537.8	175.55	0.00	20,478.7	175.70	0.00
Medical region	25	16,126.9	1.81	0.80 × 10^−2^	16,090.3	1.81	0.80 × 10^−2^	17,638.6	1.82	0.70 × 10^−2^	15,772.9	1.84	0.65 × 10^−2^
Single child	1	20,857.7	6.33	0.12 × 10^−1^	20,804.6	6.14	0.13 × 10^−1^	20,537.8	6.09	0.14 × 10^−1^	20,760.6	6.14	0.13 × 10^−1^
Years_obs_	3	20,653.1	13.66	6.73 × 10^−9^	20,688.3	13.76	5.82 × 10^−9^	20,520.3	13.68	6.54 × 10^−9^	20,314.2	13.60	7.35 × 10^−9^

Linear mixed models were used containing a gametic effect (*g*), a gametic effect as father (*g*_*s*_), a gametic effect as mother (*g*_*d*_), a maternal environmental effect (*c*), a maternal genetic effect (*m*), and a residual effect (*e*).

##### Social economic index of the mother

When considering the social economic index (SEI), analyses revealed that the effects of the mother’s SEI differed significantly for T1D with *P* values ranging from 2.56 × 10^−12^ to 1.13 × 10^−9^ across all models (Table 2; Supplementary Table S3). Although small, the largest effect (0.02; ± 3.00 × 10^−3^) was found for the intermediate group of non-manual employees (code 4). The lowest effect (− 2.00 × 10^−3^; ± 5.00 × 10^−3^) was found for professionals as well as higher civil servants and executives (code 5).

##### Medical region

**Figure 3:**
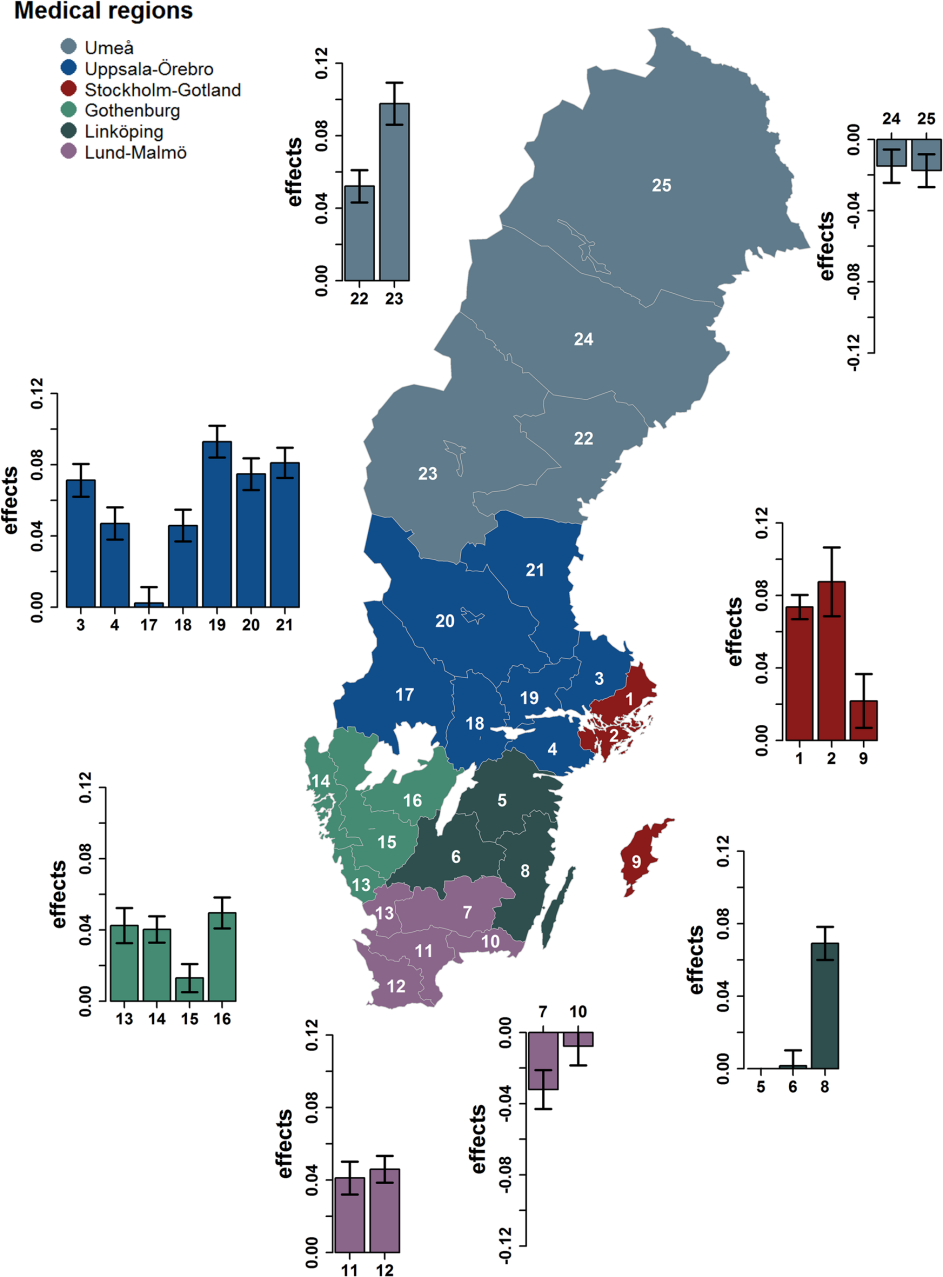
Effects of counties on the susceptibility to type 1 diabetes summarised into medical regions in Sweden. Effects were estimated using a linear mixed model that includes a random gametic effect and a random maternal environmental effect. Standard errors are indicated by error bars. Coordinates of Sweden were downloaded from https://www.scb.se/hitta-statistik/regional-statistik-och-kartor/regionala-indelningar/digitala-granser/ (accessed in November 2019) in the ArcView-shape format. Data were edited using the “readOGR” function implemented in the R-package “rgdal” version 1.4-8 (Bivand, R., Keitt, T. & Rowlingson, B. rgdal: Bindings for the 'Geospatial' Data Abstraction Library. R package version 1.4-8. (2019)) which was used in R version 3.6.1 (R Core Team (2019). R: A Language and Environment for Statistical Computing (R Foundation for Statistical Computing, Vienna, Austria)).

To investigate the effect of geographical location on T1D susceptibility, medical regions were used. Using linear and threshold models, effects differed significantly for T1D across medical regions with *P* values ranging from 2.12 × 10^−156^ to 4.60 × 10^−104^ (Table 2; Supplementary Table S3). As depicted in Fig. 3, effect sizes varied widely across Sweden.

##### Sex

A slight male skew towards T1D was observed (14,626 male vs. 12,629 female), with significantly different effects seen across all models for sex. *P* values ranged from 8.05 × 10^−225^ to 3.23 × 10^−20^ (Table 2; Supplementary Table S3). The analyses further revealed significant interactions between sex and birth year with *P* values ranging from 1.50 × 10^−50^ to 3.45 × 10^−11^ (Table 2; Supplementary Table S3). Minimal changes were found for estimates across models. The effect of male sex on T1D increased proportionally with birth year starting in 1965, reaching its highest point in the late 1970s, and declined until no interactions could be observed in 1990 (Supplementary Fig. S1).

#### Rheumatoid arthritis

##### Birth year

Ranging from 1939 to 2007, the effects of birth year differed significantly across all models with *P* values ranging from 3.34 × 10^−265^ to 2.64 × 10^−24^ (Table 2; Supplementary Table S3). Negative effects were observed from 1939 with the lowest point obtained in 1958. Since then, RA susceptibility has increased with a positive effect being observed in 1963; a trend that continued until the end of the 1970s. The trend remained constant for approximately 10 years, with a slight increase noticeable towards the end of the 1980s. Increasing standard errors must however be noted (Fig. 2). While a similar trend was observed for the threshold models (data not shown), effects increased in 1974 and remained constant before decreasing in 1991. Large standard errors were observed for effects after 1973.

##### SEI of individual

The SEIs of individuals significantly differed for RA with an average *P* value of 1.66 × 10^−9^ for linear models and *P* = 0.01 for threshold models (Table 2; Supplementary Table S3). Effect estimates varied little across models. The largest effect (0.11; ± 0.03) was found for unskilled or semi-skilled workers, while the lowest effect (0.02; ± 8.00 × 10^−3^) was observed for foremen in industrial production and assistant non-manual employees.

##### SEI of the mother

Maternal SEIs had a small but significant effect on RA susceptibility in offspring under both the linear and threshold models. *P* values ranged from 0.01 to 0.02 (Table 2; Supplementary Table S3). Effects varied little across models with similar estimates being calculated. The lowest effect was found for foremen in industrial production and assistant non-manual employees (− 0.03; ± 0.01), followed by skilled manual workers (− 0.01; ± 0.01). Except for the unknown SEI group, the highest effect was found for the group of professionals as well as higher civil servants and executives (4.00 × 10^−3^; ± 0.01).

##### Number of offspring

**Figure 4:**
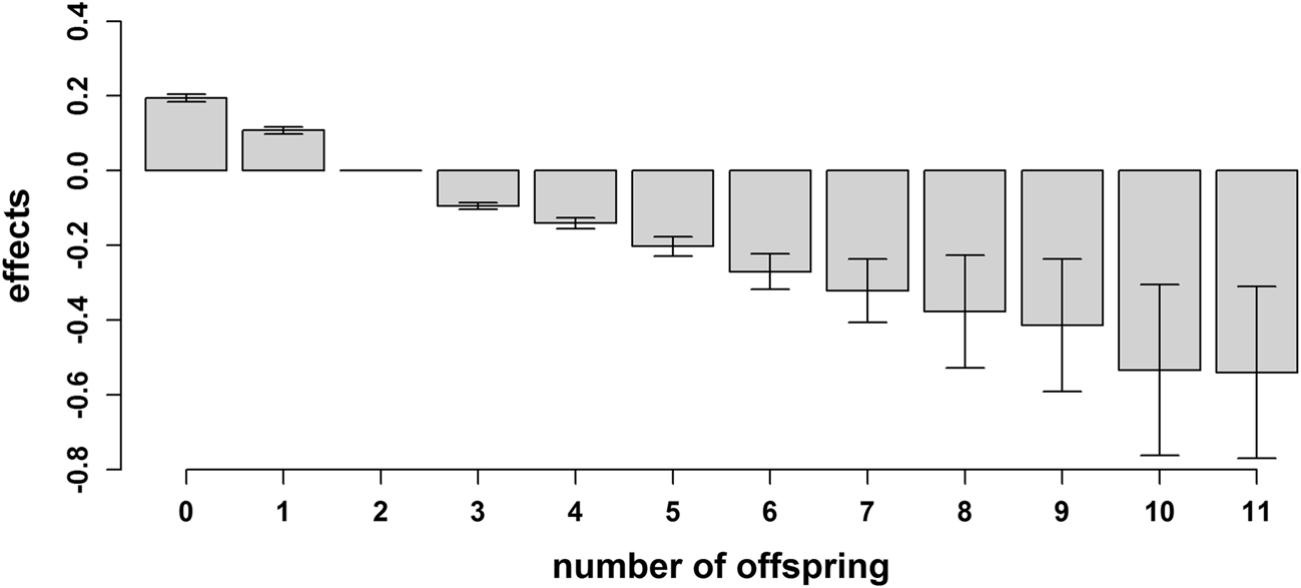
Effects of the number of offspring on the susceptibility to rheumatoid arthritis estimated using a linear mixed model that includes a random gametic, a random maternal environmental, and a random maternal genetic effect. The standard errors are indicated by error bars.

In the dataset, women had an average number of 1.94 children (ranging from zero to 11 children; *sd* = 1.25), while men had an average number of 2.04 children (ranging from zero to 11 children; *sd* = 1.37). The number of offspring affected RA development significantly across all models (*P* < 1.00 × 10^−3^). An inverse and almost linear relationship between RA susceptibility and the number of children is depicted in Fig. 4. While effects greater than zero were estimated for individuals with zero, one or two children, decreasing effects were observed below zero for individuals with more than two children.

##### Medical region

Medical regions, serving as the proxy for residential and geographic location, differed significantly in their impact on RA with *P* values ranging from 0.70 × 10^−2^ to 0.01 across all models (Table 2; Supplementary Table S3). Effect sizes varied widely across Sweden and were generally small with large standard errors (Supplementary Fig. S2).

##### Single child

We found that being a single child or having siblings made a significant difference regarding RA susceptibility with *P* values ranging from 0.01 to 0.02 across the models (Table 2; Supplementary Table S3). For the linear models, the mean estimated effect of being a single child was − 0.02 (± 9.00 × 10^−3^), while an effect of − 0.15 (± 0.06) was observed under the threshold models.

##### Sex

The incidence of RA was considerably higher in women than in men with 11,442 female and 4,408 male cases, respectively. Sex effects were significantly different with *P* values ranging from 6.86 × 10^−149^ to 6.61 × 10^−101^ across models (Table 2; Supplementary Table S3). Interactions between sex and birth year were significant only under the linear models (*P* = 0.03; *P* = 1.00 in the threshold models). No clear trend was visible for interaction effects amongst male patients (Supplementary Fig. S1). Significant interactions between sex and SEI (average *P* value of 4.56 × 10^−6^) as well as between sex and the number of offspring were observed (average *P* value of 2.55 × 10^−15^). The latter interaction was not significant using threshold models (average *P* value of 0.48; Supplementary Table S3).

### Linear versus threshold model

Firstly, the concordance across the linear and threshold model results were investigated by comparing the predicted genetic values under *Mendelian model 1*. High Pearson correlation coefficients were obtained with *r* = 0.99 for both T1D and RA. The linear relationships are shown in Supplementary Fig. S3. Secondly, threshold genetic values were fitted using the linear genetic values as independent variables. These results and their respective residual values are shown in Supplementary Fig. S3. The residual variation was fairly constant with some outliers observed over the entire range for T1D and RA.

## Discussion

### Parent-of-origin effects

Our finding that imprinting did not seem to affect T1D susceptibility supported previous findings by McCarthy et al. (1991), who analysed the importance of imprinting in an epidemiological study of clinical data from the Children’s Hospital of Pittsburgh IDDM Registry in Pennsylvania, USA^29^. They rejected the imprinting hypothesis and suggested that other genetic and environmental factors may have caused disease occurrence^29^. In addition, Guo and Tuomilehto (2002) stated that the male preponderance in T1D prevalence, fecundity differences, misclassification of T1D and birth order effects could have led to a higher T1D-prevalence in children of T1D-affected fathers^30^. In contrast, a genome-wide association study of European T1D patients showed that an imprinted T1D-associated locus was located within the maternally expressed *MEG3* gene^31^.

With regard to RA our findings are consistent with observations made by Zhou et al*.* (2007), who found that imprinting is unlikely to affect the susceptibility to RA^34^. In most tissues, the *IGF2* (insulin-like growth factor 2) gene is only paternally expressed, i.e. the maternal allele is imprinted. Martin-Trujillo et al*.* (2010) found an increased expression of *IGF2* in a subset of RA fibroblast-like synoviocytes^33^. This cell type forms the synovial intimal lining and contributes to cartilage destruction and synovial inflammation^49^. The authors reported that *IGF2*-linked “loss of imprinting” was responsible for the increased expression that contributed to the autonomous growth of RA fibroblast-like synoviocytes^33^. These findings demonstrated the effects of partially imprinted loci on RA susceptibility.

In our study, the hypothesis that imprinted loci were the major cause influencing T1D and RA susceptibility was rejected. However, where a small number of fully or partially imprinted genes with small or moderate effect sizes does exist, the relative imprinting variance (ratio between the imprinting variance and the total additive genetic variance) is expected to be small. Although the *imprinting model* considers all kinds of imprinting^17,36,37^, our study may have been underpowered and therefore unable to obtain statistical significance. Power to detect significant imprinting variances depends on both the *h*^2^ value of a trait and relative imprinting variance. In this study, heritability estimates for T1D and RA were comparatively small at 19.00% and 10.00%, respectively. However, *h*^2^ estimates vary widely in literature; T1D estimates range between 40 and 92%^3^ while RA estimates range between 12 and 60%^11–13^. Estimates also depend on the underlying data or the method used in the analysis. For example, twin studies result in broad sense heritability estimates where epigenetics are not taken into account. This increases the risk of inflated estimates^3,50^. Furthermore, sample size and pedigree information affect the ability to estimate genetic parameters and thus the power to detect significance. The depth of the pedigrees was theoretically sufficient to derive imprinting variances, but coancestry information between the maternal and paternal gametes of individuals with RA phenotypes would be a requirement for imprinting variance components analyses. In this context, the availability of genealogy databases in combination with genotypic data would increase the traceability of coancestries. Examples of such databases include the Utah Population database and a number of reliable Icelandic databases^51,52^. The availability of population-scale family trees^53^ would further allow the determination of the parental origin of alleles^54^ and generally enable large scale human population studies on epidemiological history^55^. Overall, the reliability of data and its impact on T1D and RA diagnoses must be discussed. According to Ludvigsson et al. (2011) the ratio of correct diagnoses for RA in the Swedish Hospital Discharge Register is 93.5% and 87.1% with and without lymphoma, respectively. In this study, no distinction was made between T1D and T2D from ICD-7 through ICD-9. T1D was therefore defined according the age at first hospitalisation as being not older than 20 years. For T1D and T2D 79% of cases were correctly diagnosed. The Swedish Hospital Discharge Register has provided complete national coverage since 1987 with more than 99% of all somatic hospital discharges currently registered^56^.

The replicability of the results of this study depends on the available data basis. Our results are based on data collected until 2007 (RA) and 2012 (T1D). The constant upgrade of the Swedish Hospital Discharge Register, supported by an increasing digitization of data collections, will further improve the size of good quality data and increase the pedigree depth so that higher power to detect significance can be expected when fitting our methods. Furthermore, while disease incidences in the data may not reflect the population incidences, the extension of data will lead to an accumulation of information on family affiliations as well as on the occurrence of the disorders across generations. This will improve the efficiency of estimating heritability in general and the separation of variance components in particular.

With regard to the impact of the mother, we found highly significant maternally derived environmental effects on T1D susceptibility. This finding is supported by Hirschhorn (2003) who stated that there are convincing data that non-genetic factors, such as environmental factors in early childhood, play a role in T1D susceptibility^7^, while Nisticò et al. (2012) found significant effects of the shared environment on T1D susceptibility in an Italian twin cohort study^57^. Maternal environmental effects are expected to especially affect an individual in utero, perinatally, or during early childhood (familial environment)^58^. Factors such as early exposure to cow milk and cereals or a shortened duration of breastfeeding have been mentioned in this context^58^. In addition, we found the mother’s SEI to have a significant effect on T1D susceptibility in her offspring. It should however be considered that the SEI of a mother is in itself influenced by her environment. Examples include her husband’s SEI or other life circumstances. Apart from indications for the importance of maternal environmental effects, corresponding variance component analyses in full-sib populations could indicate the existence of an autosomal dominant inheritance pattern. However, based on findings from studies in Finland and the University of Southern California, Jerram and Leslie (2017) concluded that T1D susceptibility is unlikely to be affected by autosomal dominant genes^59^.

With regard to RA, the maternal genetic variance was found to be significant when maternal environmental effects were not part of the model. Moreover, results indicated an inflation of both, the additive genetic and the maternal environmental variance by maternal genetic effects if ignored in the models. The threshold model results underline these findings. Maternal genetic effects on RA susceptibility have not been reported before. However, as sex-linked effects such as X-chromosomal and mitochondrial effects were not considered in the analysis, an inflation of the maternal genetic variance cannot be excluded. The role of the X-chromosome for the development of RA has previously been discussed; however, these studies have focused on the impact of skewed X-chromosomal inactivation in the context of sex differences in RA susceptibility^60,61^. In addition, the existence of maternal environmental effects on RA susceptibility cannot be excluded. In utero effects, which include maternal smoking^62^ or protective effects of maternal non-inherited HLA-antigens^63^, breast-feeding in perinatal life^64^, or hygiene standards during postnatal development^44^ have been reported. This study found that RA development was affected by the mother’s SEI. To conclude, while their relative importance could not clearly be quantified, both maternal genetic and environmental effects are indicated in RA susceptibility. Furthermore, while the ability to estimate covariances depends on the population structure^65^, the existence of covariances between additive and maternal genetic effects is nevertheless possible given that the *h*^2^ value was reduced to zero when a maternal genetic effect was added to the various models.

Regardless, our study clearly indicated the significance of maternal effects on the development of T1D and RA. This was important as some of the contributory factors could be modified, possibly leading to the prevention of disease or treatment interventions^66^.

### Environmental and sex effects

The development of T1D and RA were found to be significantly different over the last century when considering the effects of the year of birth. With regard to T1D, Gale (2002) stated that an increase in the incidence thereof over the second half of the twentieth century within a genetically relatively stable population would imply that environmental factors play a role in its etiology^67^. Hence, birth year effects could be attributed to environmental factors which have changed during the last century. There is a reason to suspect that these factors are linked to adjustments in living conditions which are, among others, affected by the economic state of a country. The Swedish economy has been characterised by a steady acceleration in economic growth with decelerations observed during the 1970s and early 1980s^68^. Increased disposable incomes are usually associated with improved living conditions. That living conditions can have an effect on T1D susceptibility is supported by our finding that the SEI of the mother, and thus the environment she provides for her children, significantly affects their likelihood of being diagnosed with T1D. In earlier studies, the susceptibility to T1D has also been shown to be associated with increased, lifestyle-associated linear growth and obesity^69^. While T1D susceptibility varied between regions, medical and the presumed corresponding residential regions were found to significantly affect the likelihood of being diagnosed with this condition. This finding is supported by Tzaneva et al. (2001), who reported that the onset of T1D is strongly dependent on the area of residence^70^. Nevertheless, as observed in Fig. 3, the varying effects associated with the medical regions and year of birth might also be due to the periodic and regional variation in the construction of the Swedish Hospital Discharge Register and Outpatient Register. While the Swedish Hospital Discharge Register was founded in 1964 in six Swedish counties mainly located in the Uppsala region, its nationwide launch was only in 1986. Notably, the Swedish Hospital Discharge Register is now almost 100% with lower coverage of hospital-based outpatient care (approximately 80%)^56^.

As rheumatoid arthritis usually occurs later in life, it cannot accurately be determined which environmental factors have operated during a lifetime. There is evidence that an immune system can be permanently modified by environmental factors at an early age, with growth, nutrition and infectious exposure already having activated the immune system before disease onset^44^. This was supported by our novel finding that the SEI of the mother significantly affected RA susceptibility in children and that the SEI of the individual affected RA susceptibility later in life. The latter effect is also influenced by environmental factors such as the intake of oral contraceptives^71^. In addition, an inverse relationship between the total number of children and RA susceptibility was found. Pregnancy has been reported to typically ameliorate symptoms of RA^72^ and breast feeding is known to decrease RA susceptibility^64^. With regard to men, causal effects remain to be discussed. It should be noted that while the effect of the number of children on RA susceptibility was not investigated separately in women and men, significant interactions between sex and the number of children underline existing sex-associated differences. The difficulty in determining when environmental triggers occurred in individual life times may be reflected in the challenging endeavor of estimating the effects of medical regions on disease susceptibility. No information was available for how long individuals have resided in the analysed regions, potentially explaining the small effects and large standard errors. However, as for T1D, the periodic and regional variation in the data collection might also be a reason for this^56^. Nevertheless, geographical variation in RA incidence has previously been reported. Indications were found for the lower incidence rates in South European countries compared to North American and north European countries^73,74^.

In the context of sex differences in T1D and RA susceptibility, effects of the X-chromosome, sex hormones, sex-specific behavior, and sex-specific differences in body composition and structure have been discussed^32,60,61,75,76^. Our study confirmed that there was a significant sex effect in the development of both disorders. Moreover, especially for T1D, significant interactions between sex and birth year were found. This raises the question whether environmental effects preferentially interact with either sex. Based on the hypothesis that the prevalence of T1D amongst males increases proportionally in relation to disease incidence when the underlying environmental causes preferentially affect males, Gale and Gillespie (2001) reviewed sex ratios in T1D-incidence in multiple populations. They found that, although disease incidence increased, the sex ratio does not change and the trend towards male cases is specific for some populations^75^. They concluded that environmental effects do not interact with males over females. In our study, the effect of sex on T1D was only visible from 1960 to the mid-1980s.

### Linear versus threshold model

One difficulty in this study was that the affection status was measured as a categorical trait with binomial distributions. Therefore, the linear models are not appropriate to analyze this data type. Nevertheless, statistical significance of genetic parameters could only be tested using the REML log-likelihood of linear models via RLRT. When a threshold model is used, the ASReml-package employs an approximate likelihood (penalised quasi-likelihood) that cannot be used to test differences^77^. There are currently no alternatives to the ASReml-package for our specific imprinting analysis as it is the only package that allows setting an appropriate correlation between the two parental gametic effects. This function ensures equivalence between the *Mendelian* and *imprinting models*. Equivalence is needed to perform an RLRT. Regarding the utilisation of linear and threshold models, both models generated similar results in uncovering the underlying genetic variation for T1D and RA. High correlations between the estimated genetic values indicated a fairly good fit of both models to the data.

## Conclusion

Not only was new knowledge gained on the environmental effects on T1D and RA development, the separate contributions of each POE was able to delineate the genetic and phenotypic variation in T1D and RA susceptibility for the first time. Results supported findings that imprinting was of minor importance, but confirmed the role maternal factors played in the occurrence of both diseases. The prospects of fitting complex genetic variance–covariance structures can be expected to further improve given the size of good quality epidemiological data and pedigree depth.

## Material

### General data

This study was based on Swedish population-based registries with national coverage. Registry entries were linked using each person’s unique identification number. To ensure participant confidentiality, this identification number was replaced by a serial number. The project registry was linked to the Multigeneration Register and Population Registers providing information on family relations, and SEI (in Swedish, socioekonomisk indelning), medical region and birth year, respectively. Individuals diagnosed with AIs were identified from the Swedish Hospital Discharge Register (contains data regarding hospitalisation and diagnoses in some regions since 1964 and nationwide since 1986^78^) and from the Outpatient Register since 2001. For further information see Hemminki (2001) and Hemminki et al. (2009)^79,80^.

### Type 1 diabetes

**Figure 5:**
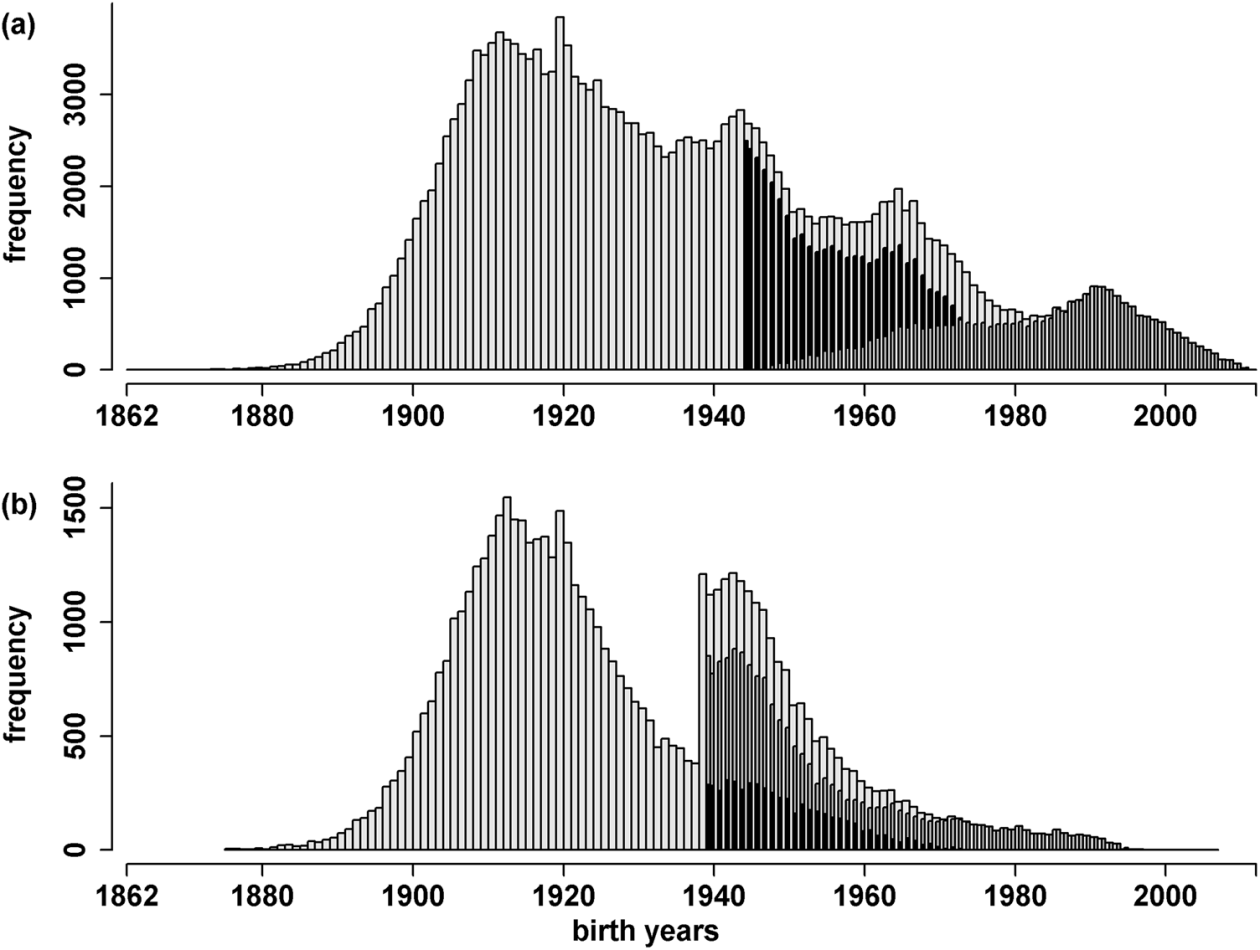
Distribution of birth years of all individuals in the pedigrees (light gray) as well as of all controls (black) and cases (gray) of type 1 diabetes mellitus (**a**) and rheumatoid arthritis (**b**).

The data selection for T1D was strict. T1D was not defined as an independent entity until the ICD-10 (International Classification of Diseases) classification system^80^. Therefore, T1D was defined through first hospitalisation until an age of 20 years in addition to the ICD codes (ICD-7 code 250, ICD-8 code 260, ICD-9 code 260 or ICD-10 code E10) to unambiguously delineate T1D from type 2 diabetes^81^. Furthermore, as AIs were recorded from 1964, it was assumed that the health status of individuals born prior to 1944 cannot be unambiguously defined. Therefore, only individuals born after 1943 were assigned a case/control status (Fig. 5). Furthermore, only individuals born in Sweden and with known maternal data were considered in the analyses. Overall 27,255 T1D patients (14,626 male and 12,629 female) and 43,856 controls (20,234 male and 23,622 female) were used for the analyses. All individuals were born between 1944 and 2012. The age at diagnosis ranged between zero and 20 years with a mean age of 11 years (*sd* = 5.16; Supplementary Fig. S4). Ancestral data was extracted from the Multigeneration Register for all patients. The complete dataset included 208,114 individuals (103,434 male and 104,680 female), with birth years ranging between 1862 and 2012. The dataset contained five generations with 111,626 founders and 23,124 families. The smallest family contained two individuals, and the largest 91 individuals. The average number of cases per family was 1.18 (the lowest number was one case per family; the highest number was 12 cases per family).

### Rheumatoid arthritis

To not erroneously assign an individual with RA to the control status, health status was only declared for individuals born after 1938, i.e. for individuals not older than 25 years in 1964 when AIs registration was initiated (Fig. 5). Cases were selected according to the following codes: ICD-7 code 722, ICD-8 codes 712.0, 712.1, and 712.3, ICD-9 code 714, and ICD-10 codes M05, M06, M08.0, and M08.2. Only individuals born in Sweden and with known maternal data were considered in the analyses. Overall 15,850 patients (4,408 male and 11,442 female) diagnosed with RA and 5,199 controls (2,358 male and 2,841 female) were used for the analyses. They were born between 1939 and 2007. The age at diagnosis ranged between zero and 73 years with a mean age of 46.29 years (*sd* = 16.65; Supplementary Fig. S4). Ancestral data was extracted from the Multigeneration Register for all patients. The generated dataset included 60,684 individuals (26,339 male and 34,345 female), with birth years ranging between 1875 and 2007. The dataset contained five generations with 37,142 founders and 15,314 families. The smallest family contained two individuals, and the largest 21 individuals. The average number of cases per family was 1.04 (the lowest number was one case per family; the highest number was six cases per family).

## Methods

### The Mendelian models

To test the significance of the imprinting variance, a nested, equivalent version of the *imprinting model* must be applied. This model predicts a reduced number of parameters. It is called the *Mendelian model* as it represents the null-hypothesis that imprinting does not exist, i.e. the two parental gametes are not expressed differently (pure Mendelian inheritance). It can be written as:

$${\varvec{Y}} = {\varvec{Xb}} + {\varvec{Z}}_{{\varvec{g}}} {\varvec{g}} + {\varvec{e}},\quad \quad \quad \quad \quad \quad \left( {Mendelian\;model\;1} \right)$$

where ***Y*** is a vector of the response variable; ***b*** is a vector of fixed effects; ***g*** is the vector of random gametic effects; ***X ***and* Z*_***g***_ are incidence matrices; and ***e*** is the vector of random residuals. They are assumed to be normally distributed with a mean of 0 and variances ***G***$\sigma_{g}^{2}$.

### Model extensions

Other POEs such as maternal genetic or maternal environmental effects have been reported to be potential nuisance factors, i.e. erroneously assumed to be maternal effects due to imprinting, if not considered within the model definitions^41^. To be able to distinguish maternal effects due to imprinting from other POEs as well as to investigate the importance of the latter, the following versions of *Mendelian model 1* were applied:

$$\begin{aligned} {\varvec{Y}} & = {\varvec{Xb}} + {\varvec{Z}}_{{\varvec{g}}} {\varvec{g}} + {\varvec{Z}}_{{\varvec{c}}} {\varvec{c}} + {\varvec{e}},\quad \quad \quad \quad \quad \quad \quad \quad \quad \quad \quad \left( {Mendelian\;model\;2} \right) \\ {\varvec{Y}} & = {\varvec{Xb}} + {\varvec{Z}}_{{\varvec{g}}} {\varvec{g}} + {\varvec{Z}}_{{\varvec{c}}} {\varvec{c}} + {\varvec{Z}}_{{\varvec{m}}} {\varvec{m}} + {\varvec{e}},\quad \quad \quad \quad \quad \quad \quad \quad \left( {Mendelian\;model\;3} \right) \\ \end{aligned}$$

where ***c ***and ***m*** are vectors of random maternal environmental and maternal genetic effects, respectively. They are assumed to be normally distributed with a mean of 0 and variances ***I***_***c***_ $\sigma_{c}^{2}$ and ***A***$\sigma_{m}^{2}$. Matrix ***I***_***c***_ is an identity matrix and ***A*** is the additive genetic relationship matrix; the incidence matrices ***Z***_***c***_, and ***Z***_***m***_ relate observations and random effects. The significance of the additional random maternal environmental effect in *Mendelian model 2* was tested by comparing the REML log-likelihood values of *Mendelian model 1* and *Mendelian model 2* using a one-sided RLRT. The RLRT is assumed to be *χ*^2^-distributed with a degree of freedom (DF) of one. The significance of the maternal genetic effect in *Mendelian model 3* was tested by comparing the REML log-likelihood values of *Mendelian model 2* and *Mendelian model 3* using a one-sided RLRT (*χ*^2^-distributed with DF = 1).

As described for T1D, the maternal environmental variance turned out to be significant. Therefore, the following *imprinting model* was used to investigate the significance of the imprinting variance in T1D:

$${\varvec{Y}} = {\varvec{Xb}} + {\varvec{Z}}_{{\varvec{s}}} {\varvec{g}}_{{\varvec{s}}} + {\varvec{Z}}_{{\varvec{d}}} {\varvec{g}}_{{\varvec{d}}} + {\varvec{Z}}_{{\varvec{c}}} {\varvec{c}} + {\varvec{e}}.$$

This model is equivalent to *Mendelian model 2* but assumes different contributions of maternal and paternal gametes to the susceptibility to T1D.

With regard to RA, the existence of maternal environmental and maternal genetic effects could not be excluded. Hence, the *imprinting model* was applied with an additional maternal environmental effect (see above) but also with an additional maternal genetic effect:

$${\varvec{Y}} = {\varvec{Xb}} + {\varvec{Z}}_{{\varvec{s}}} {\varvec{g}}_{{\varvec{s}}} + {\varvec{Z}}_{{\varvec{d}}} {\varvec{g}}_{{\varvec{d}}} + {\varvec{Z}}_{{\varvec{m}}} {\varvec{m}} + {\varvec{e}}.$$

In general, the significance of the imprinting variance was determined by comparing the REML log-likelihood value of the *imprinting model* with the REML log-likelihood outcome of the corresponding *Mendelian models* using a one-sided RLRT. The test statistic was assumed to be asymptotically distributed as a mixture of two *χ*^2^ distributions with DF = 1 and DF = 2^36–40,82^.

### Calculation of population parameters

For the *Mendelian models* the direct heritabilities were calculated as $h_{Mendel}^{2} = \sigma_{a}^{2} /\sigma_{p}^{2}$, where $\sigma_{a}^{2} = 2\sigma_{g}^{2}$ and $\sigma_{p}^{2} = \sigma_{g}^{2} + \sigma_{e}^{2}$ in *Mendelian model 1*, $\sigma_{p}^{2} = \sigma_{g}^{2} + \sigma_{e}^{2} + \sigma_{c}^{2}$ in *Mendelian model 2*, and $\sigma_{p}^{2} = \sigma_{g}^{2} + \sigma_{e}^{2} + \sigma_{c}^{2} + \sigma_{m}^{2}$ in *Mendelian model 3*. The latter two expressions of $\sigma_{p}^{2}$ were used to calculate $c_{Mendel}^{2}$ ($c_{Mendel}^{2} = \sigma_{c}^{2} /\sigma_{p}^{2}$) for *Mendelian model 2* and *Mendelian model 3*, respectively. To calculate $m_{Mendel}^{2}$ ($m_{Mendel}^{2} = \sigma_{m}^{2} /\sigma_{p}^{2}$) for *Mendelian model 3*, $\sigma_{p}^{2}$ was defined as $\sigma_{p}^{2} = \sigma_{g}^{2} + \sigma_{e}^{2} + \sigma_{c}^{2} + \sigma_{m}^{2}$.

For the *imprinting models* the direct heritabilities were calculated as $h_{imp}^{2} = \sigma_{a}^{2} /\sigma_{p}^{2}$, where $\sigma_{a}^{2} = \sigma_{gs}^{2} + \sigma_{gd}^{2}$ and $\sigma_{p}^{2} = \sigma_{gs}^{2} + \sigma_{gd}^{2} + \sigma_{e}^{2} + \sigma_{c}^{2}$ or $\sigma_{p}^{2} = \sigma_{gs}^{2} + \sigma_{gd}^{2} + \sigma_{e}^{2} + \sigma_{m}^{2}$. The first expression of $\sigma_{p}^{2}$ can be used to calculate $c_{imp}^{2}$ as $c_{imp}^{2} = \sigma_{c}^{2} /\sigma_{p}^{2}$. The latter expression of $\sigma_{p}^{2}$ can be used to calculate $m_{imp}^{2}$ ($m_{imp}^{2} = \sigma_{m}^{2} /\sigma_{p}^{2}$).

### Threshold model

As the case/control-status is denoted 1/0, the trait can be assumed binomially distributed. Hence, apart from the linear mixed models, which were needed for hypotheses testing, generalised linear mixed models (threshold models) were applied using a logit link and the pseudo-likelihood approach of Gilmour et al. (2015)^77^. The probability that an observation with index *k* belongs to class zero is:

$$\pi \left( {\eta_{k} } \right) = \exp \left( {\eta_{k} } \right)/\left[ {1 + \exp \left( {\eta_{k} } \right)} \right],$$

where – using *Mendelian model 2* as an example—the linear predictor is:

$$\eta_{k} = x_{k} \beta + z_{g,k} g + z_{c,k} c$$

and *x*_*k*_, *z*_*g,k*_, and *z*_*c,k*_ are the *k*th rows of the aforementioned incidence matrices ***X***, ***Z***_***g***_, and ***Z***_***c***_ and the vectors ***ß***, ***g*** and ***c*** are defined as described for *Mendelian model 2*.

### Fixed effects

Fixed effects included sex (2 levels; 1 = male, 2 = female), birth year (68 birth years for T1D; 57 birth years for RA), medical region (26 levels; individuals lived in 25 medical regions [26 = unknown]), and the SEI of the mother (6 levels) for which the following codes were used:

- 1 = unskilled/ semi-skilled workers
- 2 = skilled workers
- 3 = foremen in industrial production and assistant non-manual employees
- 4 = intermediate non-manual employees
- 5 = employed and self-employed professionals and higher civil servants and executives
- 6 = unknown

Note that coding differed across the various versions of the population database. Therefore, they were adjusted and equalised according to the 2012 version. The time an individual was under observation was considered by including the Legendre polynomials from the years under observation up to order three (linear, quadratic and cubic). Additional effects included in the RA model were the SEI of the individual (6 levels), whether an individual was a single child or had any siblings (2 levels; 1 = single child, 2 = siblings), and the number of offspring (12 levels; range of 0–11 children). Furthermore, interactions between sex and birth years were fitted for both disorders. Interactions between sex and SEIs, as well as sex and the number of offspring were considered for RA.

Note that due to the underrepresentation of individuals in birth year levels, individuals were shifted to the next birth year level so that at least five individuals were obtained for each level.

The significance of all fixed effects was tested using an incremental Wald *F* test implemented within the statistical software package ASReml version 4.2^77^. Note that ASReml caters for linear dependencies in the model by setting singular effects to zero^83^. Variance–covariance components were also estimated via ASReml. The R-packages “kinship2” version 1.6.4^84^ and “pedigree” version 1.4^85^ in R version 3.5.2^86^ were used to prepare the data.

Coordinates of Sweden used for Fig. 3 and Supplementary Fig. S2 were downloaded from https://www.scb.se/hitta-statistik/regional-statistik-och-kartor/regionala-indelningar/digitala-granser/ (accessed in November 2019) in the ArcView-shape format. Data were edited using the “readOGR” function implemented in the R-package “rgdal” version 1.4–8^87^ which was used in R version 3.6.1^88^.

## Supplementary information

Supplementary Information 1.

Supplementary Information 2.

## Data Availability

Individual-based data for research purposes is protected by strict confidentiality protections imposed by the Swedish Government and applied by Statistics Sweden, but can be made available for research after an ethical review and a review by both Statistics Sweden and the Swedish National Board of Health and Welfare. The Swedish National Board of Health and Welfare may be contacted for data access using the following link: https://www.socialstyrelsen.se/statistik-och-data/statistik/.
