# Supplementary material for: Genomic imprinting analyses identify maternal effects as a cause of phenotypic variability in type 1 diabetes and rheumatoid arthritis
Source: Sci Rep. 2020 Jul 14;10:11562. doi: 10.1038/s41598-020-68212-x (PMC7360775; doi:10.1038/s41598-020-68212-x)
Supplement: Supplementary file 1 — Supplementary Information 1. [file 41598_2020_68212_MOESM1_ESM.pdf]

Title: Genomic imprinting analyses identify maternal effects as a cause of phenotypic variability in type 1 diabetes and rheumatoid arthritis

Authors: Inga Blunk[1], Hauke Thomsen[2,3], Norbert Reinsch[1], Manfred Mayer[1], Asta Försti[2,4,5,6], Jan Sundquist[4,7,8], Kristina Sundquist[4,7,8], Kari Hemminki[2,4,9]

Author affiliations:

[1] Institute of Genetics and Biometry, Leibniz Institute for Farm Animal Biology (FBN), Dummerstorf, Germany

[2] Division of Molecular Genetic Epidemiology, German Cancer Research Centre (DKFZ), Heidelberg, Germany

[3] GeneWerk GmbH, Heidelberg, Germany

[4] Center for Primary Health Care Research, Lund University, Malmö, Sweden

[5] Hopp Children's Cancer Center (KiTZ), Heidelberg, Germany

[6] Division of Pediatric Neurooncology, German Cancer Research Center (DKFZ), German Cancer Consortium (DKTK), Heidelberg, Germany

[7] Department of Family Medicine and Community Health, Department of Population Health Science and Policy, Icahn School of Medicine at Mount Sinai, New York, USA

[8] Center for Community-based Healthcare Research and Education (CoHRE), Department of Functional Pathology, School of Medicine, Shimane University, Japan

[9] Faculty of Medicine and Biomedical Center in Pilsen, Charles University in Prague, Pilsen, Czech Republic

Supplementary figures

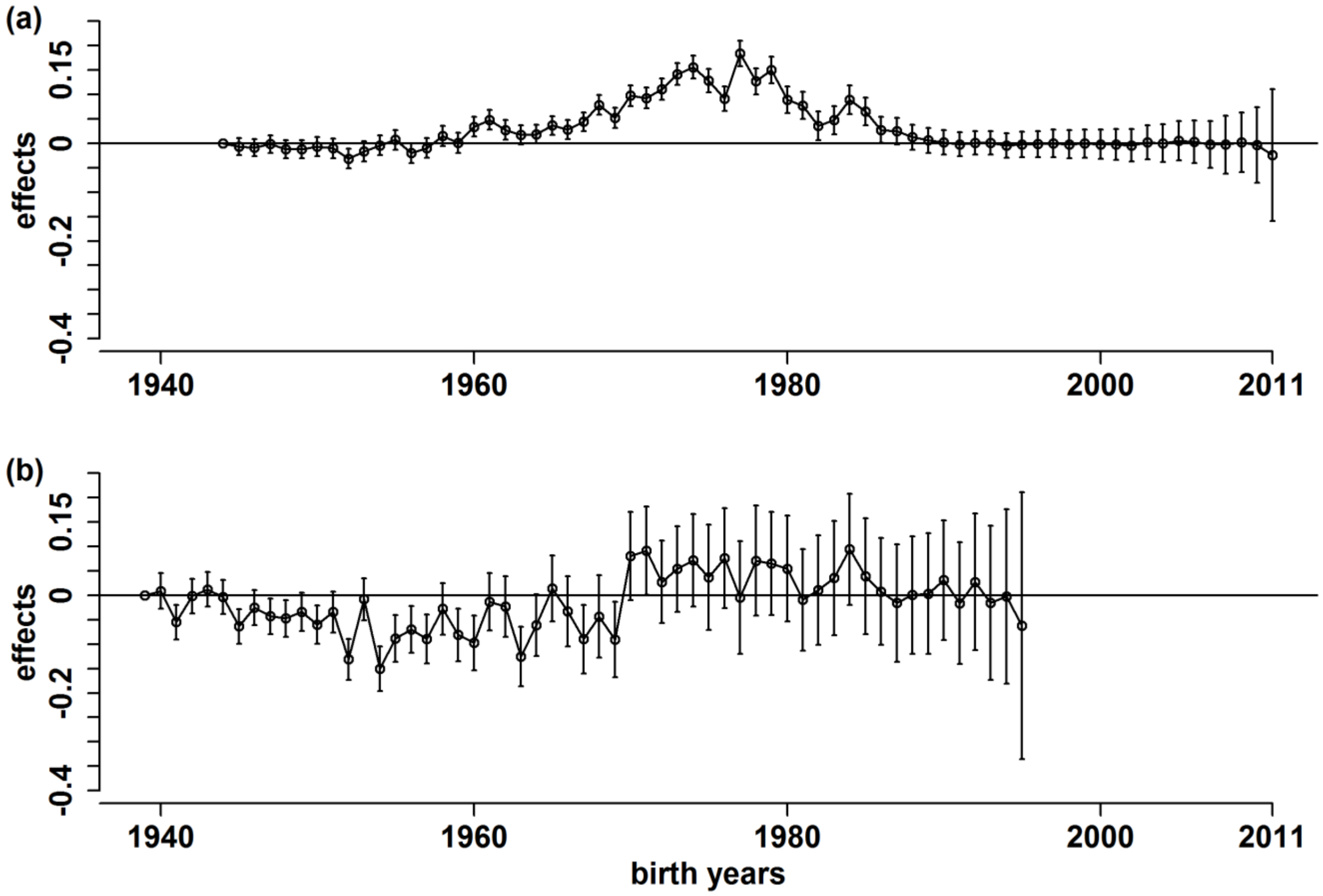

Supplementary Figure S1 Effects of male sex depending on the effect of birth year on the susceptibility to type 1 diabetes (a) and rheumatoid arthritis (b). For type 1 diabetes, interactions were estimated using a linear mixed model that includes a random gametic effect and a random maternal environmental effect. For rheumatoid arthritis a random maternal genetic effect was added. The standard errors are indicated by error bars.

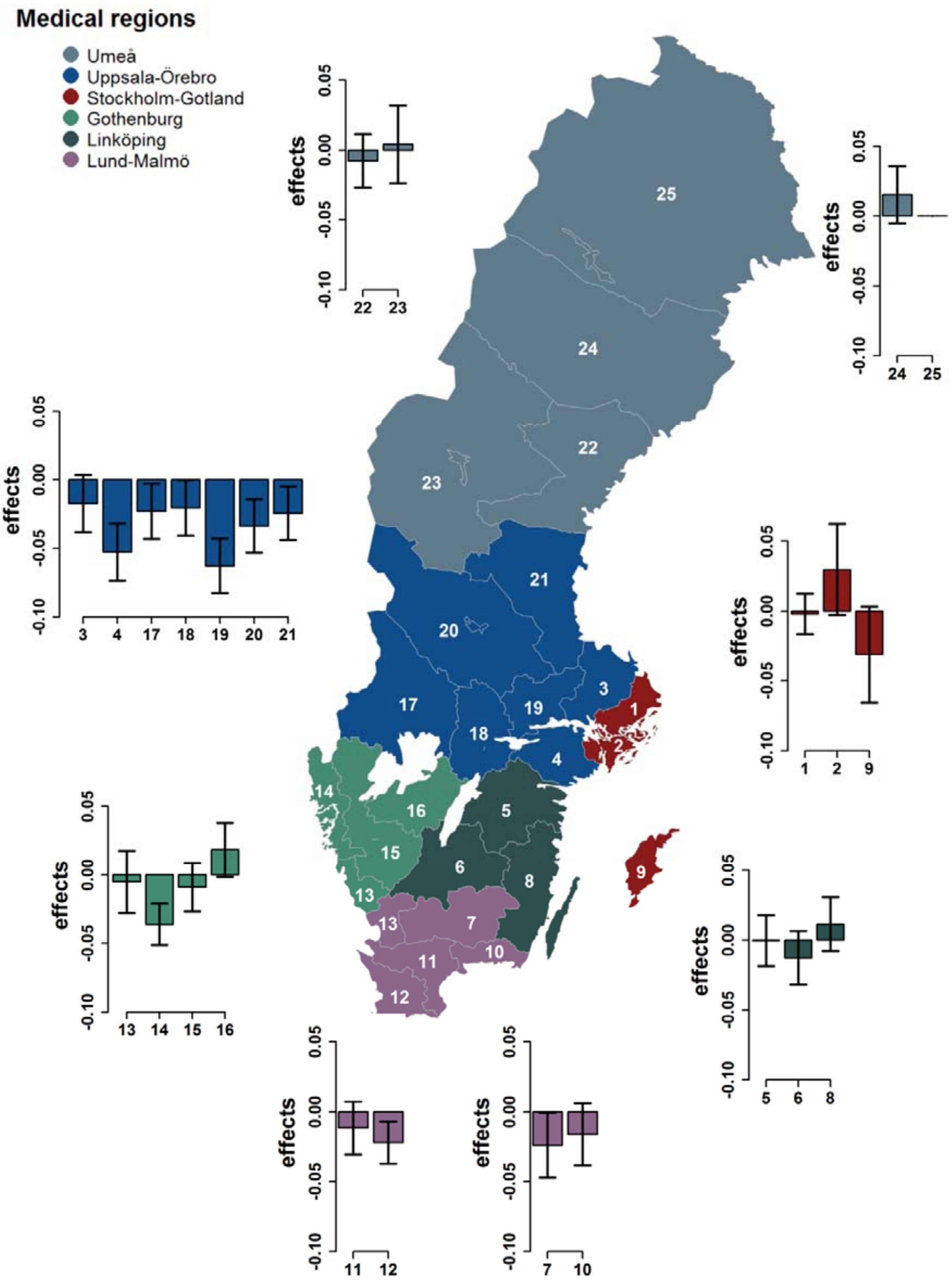

Supplementary Figure S2 Effects of counties on the susceptibility to rheumatoid arthritis summarized into medical regions in Sweden. Effects were estimated using a linear mixed model that includes a random gametic effect and a random maternal genetic effect. Standard errors are indicated by error bars. Coordinates of Sweden were

downloaded from https://www.scb.se/hitta-statistik/regional-statistik-och-kartor/regionala-indelningar/digitala-granser/ (accessed in November 2019) in the ArcView-shape format. Data were edited using the “readOGR” function implemented in the R-package “rgdal” version 1.4-8 [1] which was used in R version 3.6.1 [2].

[1] Bivand, R., Keitt, T. & Rowlingson, B. rgdal: Bindings for the 'Geospatial' Data Abstraction Library. R package version 1.4-8. (2019).

[2] R Core Team (2019). R: A Language and Environment for Statistical Computing (R Foundation for Statistical Computing, Vienna, Austria).

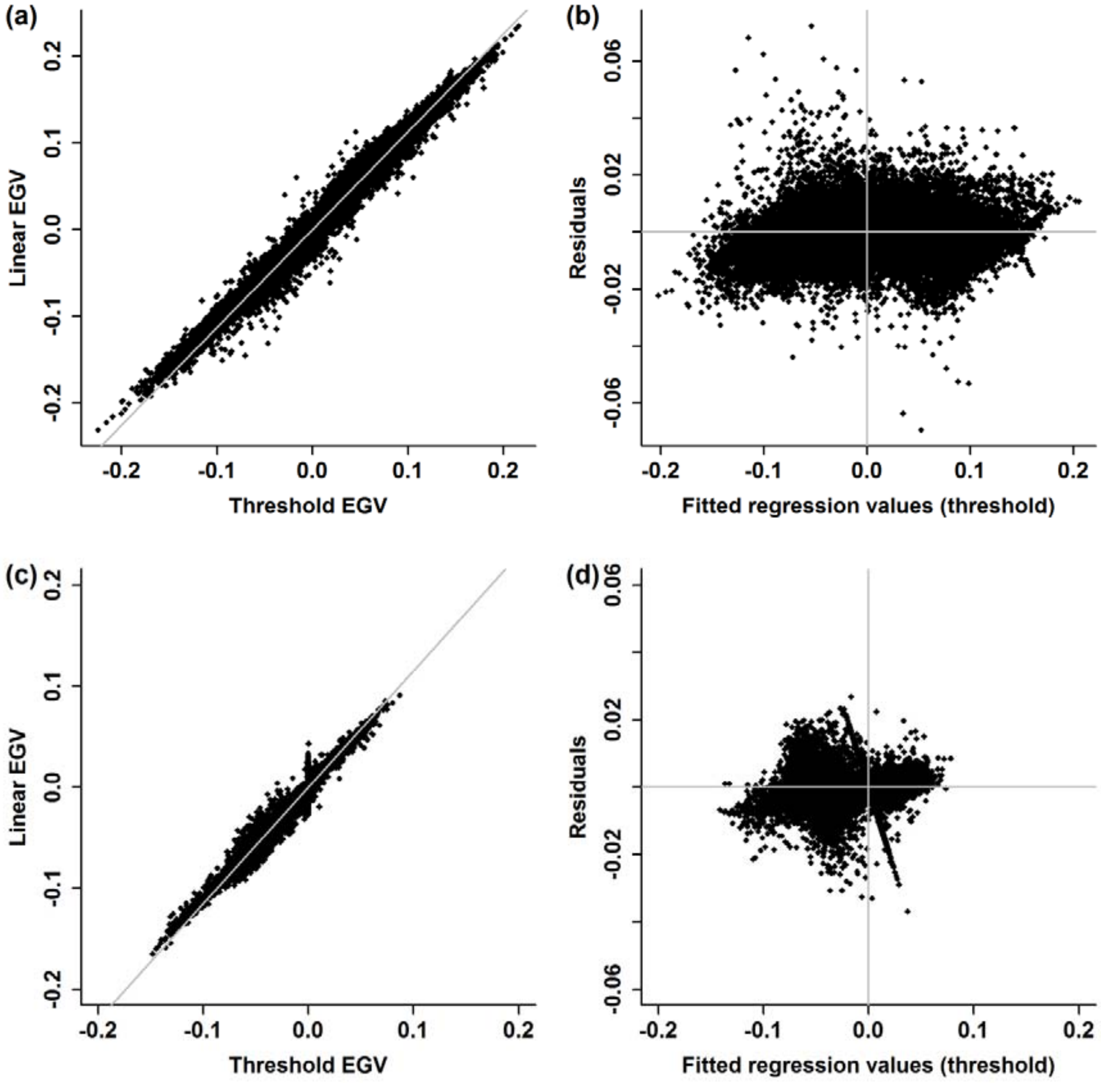

Supplementary Figure S3 Correlation between genetic values (EGVs, left) estimated using a linear (linear EGV) and a threshold model (threshold EGV). The threshold EGVs were fitted using the linear EGVs as independent variables with respect to their residuals (right) for type 1 diabetes (a, b) and rheumatoid arthritis (c, d).

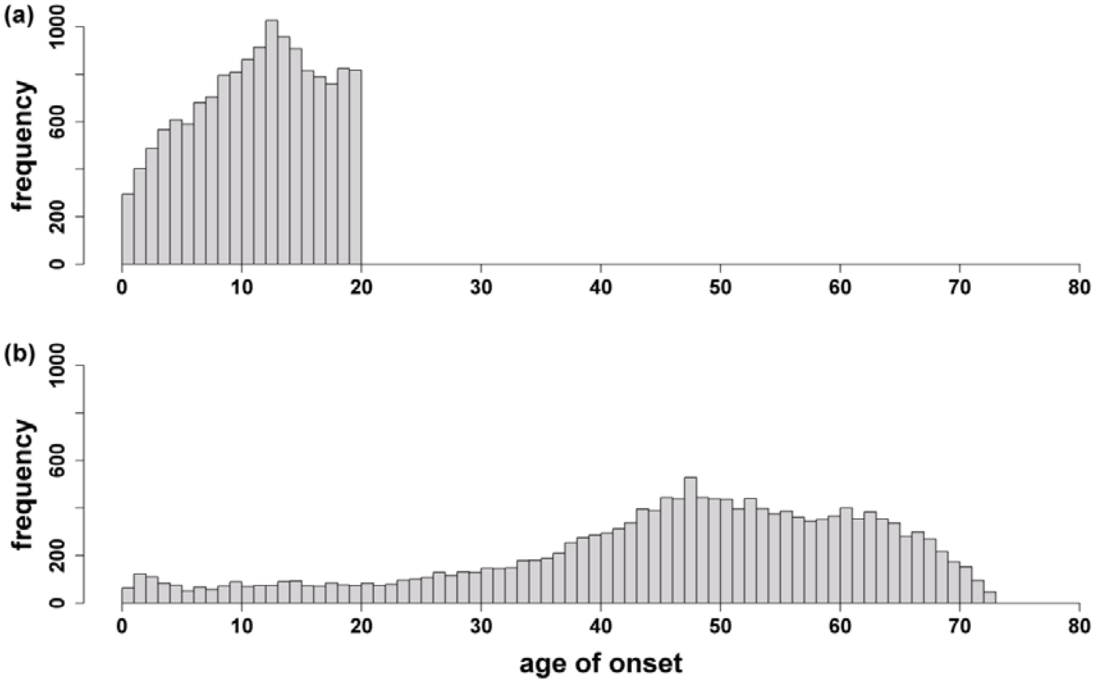

Supplementary Figure S4 Distribution of age at diagnosis with type 1 diabetes (a) and rheumatoid arthritis (b).

Supplementary tables

Supplementary Table S1. Variance and covariance components estimated for type 1 diabetes using a linear (Lin) and threshold (Thr) model containing a gametic effect (g), a gametic effect as father (g_s), a gametic effect as mother (g_d), a maternal environmental effect (c), a maternal genetic effect (m), and a residual effect (e). Standard errors are in parentheses

	Mendelian model 1		*Mendelian model 2*				*Mendelian model 3*		*imprinting model*	
Lin	$g + e$ [a]		$g + c + e$ [b]		$g + m + e$ [c]		$g + c + m + e$		$g_s + g_d + c + e$	
σ_g^2	0.82×10^{-2}	($\pm 0.5 \times 10^{-3}$)	0.46×10^{-2}	($\pm 0.6 \times 10^{-3}$)	0.11×10^{-2}	($\pm 0.8 \times 10^{-3}$)	0.46×10^{-2}	($\pm 0.6 \times 10^{-3}$)		
σ_{gs}^2									0.13×10^{-1}	($\pm 0.5 \times 10^{-2}$)
σ_{gsd}									-0.32×10^{-2}	($\pm 0.5 \times 10^{-2}$)
σ_{gd}^2									0.11×10^{-1}	($\pm 0.5 \times 10^{-2}$)
σ_a^2	0.16×10^{-1}	($\pm 0.1 \times 10^{-2}$)	0.91×10^{-2}	($\pm 0.1 \times 10^{-2}$)	0.21×10^{-2}	($\pm 0.2 \times 10^{-2}$)	0.91×10^{-2}	($\pm 0.1 \times 10^{-2}$)	0.24×10^{-1}	($\pm 0.1 \times 10^{-1}$)
σ_c^2			0.17×10^{-1}	($\pm 0.2 \times 10^{-2}$)			0.17×10^{-1}	($\pm 0.2 \times 10^{-2}$)	0.10×10^{-1}	($\pm 0.5 \times 10^{-2}$)
σ_m^2					0.16×10^{-1}	($\pm 0.2 \times 10^{-2}$)	0.10×10^{-7}	(± 0.0)		
σ_e^2	0.80×10^{-1}	($\pm 0.1 \times 10^{-2}$)	0.70×10^{-1}	($\pm 0.2 \times 10^{-2}$)	0.78×10^{-1}	($\pm 0.1 \times 10^{-2}$)	0.70×10^{-1}	($\pm 0.2 \times 10^{-2}$)	0.62×10^{-1}	($\pm 0.6 \times 10^{-2}$)
σ_p^2	0.88×10^{-1}	($\pm 0.7 \times 10^{-3}$)	0.91×10^{-1}	($\pm 0.7 \times 10^{-3}$)	0.95×10^{-1}	($\pm 0.9 \times 10^{-3}$)	0.91×10^{-1}	($\pm 0.7 \times 10^{-3}$)	0.96×10^{-1}	($\pm 0.5 \times 10^{-3}$)
h_{men}^2	0.19	($\pm 0.1 \times 10^{-1}$)	0.10	($\pm 0.1 \times 10^{-1}$)	0.23×10^{-1}	($\pm 0.2 \times 10^{-1}$)	0.10	($\pm 0.1 \times 10^{-1}$)		
h_{imp}^2									0.25	(± 0.1)
c_{men}^2			0.19	($\pm 0.2 \times 10^{-1}$)			0.19	($\pm 0.2 \times 10^{-1}$)		
c_{imp}^2									0.10	($\pm 0.5 \times 10^{-1}$)
m^2					0.17	($\pm 0.2 \times 10^{-1}$)	0.00	(± 0.0)		
P value			1.60×10^{-24} [a]		2.89×10^{-16} [a]		1.00 [b]		0.18 [b]	
							8.77×10^{-10} [c]			
L	47,228.67		47,280.90		47,262.11		47,280.90		47,282.60	
Thr	$g + e$		$g + c + e$		$g + m + e$		$g + c + m + e$		$g_s + g_d + c + e$	
σ_g^2	0.73×10^{-1}	($\pm 0.2 \times 10^{-1}$)	0.71×10^{-7}	(± 0.0)	0.83×10^{-7}	(± 0.0)	0.45×10^{-8}	(± 0.0)		

σ^2_{gs}									nc	nc
σ_{gsd}									nc	nc
σ^2_{gd}									nc	nc
σ^2_a	0.15	(±0.4 × 10^{-1})	0.14 × 10^{-6}	(±0.0)	0.17 × 10^{-6}	(±0.0)	0.90 × 10^{-8}	(±0.0)	nc	nc
σ^2_c			0.16	(±0.4 × 10^{-1})			0.15 × 10^{-6}	(±0.0)	nc	nc
σ^2_m					0.18	(±0.4 × 10^{-1})	0.18	(±0.4 × 10^{-1})		
σ^2_e	3.28		3.28		3.28		3.28		nc	nc
σ^2_p	3.35	(±0.2 × 10^{-1})	3.44	(±0.4 × 10^{-1})	3.46	(±0.4 × 10^{-1})	3.47	(±0.4 × 10^{-1})	nc	nc
h^2_{men}	0.44 × 10^{-1}	(±0.1 × 10^{-1})	0.00	(±0.0)	0.00	(±0.0)	0.00	(±0.0)		
h^2_{imp}									nc	nc
c^2_{men}			0.47 × 10^{-1}	(±0.1 × 10^{-1})			0.00	(±0.0)		
c^2_{imp}									nc	nc
m^2					0.52 × 10^{-1}	(±0.1 × 10^{-1})	0.52 × 10^{-1}	(±0.1 × 10^{-1})		
L_{ps}	–164,226.00		–163,990.99		–163,719.17		–163,719.17		nc	

σ^2_g = gametic variance; σ^2_{gs} = gametic variance as father; σ^2_{gd} = gametic variance as mother; σ_{gsd} = covariance; σ^2_a = additive genetic variance; σ^2_c = permanent maternal environmental variance; σ^2_m = maternal genetic variance; σ^2_e = residual variance; σ^2_p = phenotypic variance; h^2_{men} = heritability calculated with *Mendelian model*; h^2_{imp} = heritability calculated with *imprinting model*; c^2 = relative maternal environmental variance; m^2 = relative maternal genetic variance; L = logarithm of restricted estimated maximum likelihood (REML); L_{ps} = logarithm of pseudo likelihood generated according to pseudo likelihood approach by Gilmour *et al*. (2009); indices *a*, *b* and *c* indicate which models were compared in a REML log-likelihood ratio test generating *P* values; nc = not converged.

Supplementary Table S2. Variance and covariance components estimated for rheumatoid arthritis using a linear (Lin) and threshold (Thr) model containing a gametic effect (g), a gametic effect as father (g_s), a gametic effect as mother (g_d), a maternal environmental effect (c), a maternal genetic effect (m), and a residual effect (e). Standard errors are in parentheses

	Mendelian model 1	*Mendelian model 2*		*Mendelian model 3*	*imprinting model*	*imprinting model*
Lin	$g + e$ [a]	$g + c + e$ [b]	$g + m + e$ [c]	$g + c + m + e$	$g_s + g_d + c + e$	$g_s + g_d + m + e$
σ^2_g	0.78×10^{-2} $(\pm 0.2 \times 10^{-2})$	0.64×10^{-2} $(\pm 0.2 \times 10^{-2})$	0.73×10^{-7} (± 0.0)	0.49×10^{-7} (± 0.0)		
σ^2_{gs}					0.13×10^{-1} $(\pm 0.1 \times 10^{-1})$	0.14×10^{-2} $(\pm 0.3 \times 10^{-1})$
σ_{gsd}					-0.23×10^{-1} $(\pm 0.1 \times 10^{-1})$	0.34×10^{-3} $(\pm 0.3 \times 10^{-1})$
σ^2_{gd}					0.46×10^{-1} $(\pm 0.1 \times 10^{-1})$	0.14×10^{-2} $(\pm 0.7 \times 10^{-1})$
σ^2_a	0.16×10^{-1} $(\pm 0.5 \times 10^{-2})$	0.13×10^{-1} $(\pm 0.5 \times 10^{-2})$	0.15×10^{-6} $(\pm 0.6 \times 10^{-8})$	0.97×10^{-7} $(\pm 0.7 \times 10^{-8})$	0.60×10^{-1} $(\pm 0.2 \times 10^{-1})$	0.28×10^{-2} (± 0.1)
σ^2_c		0.23×10^{-1} $(\pm 0.9 \times 10^{-2})$		0.77×10^{-2} $(\pm 0.1 \times 10^{-1})$	0.19×10^{-7} (± 0.0)	
σ^2_m			0.25×10^{-1} $(\pm 0.5 \times 10^{-2})$	0.21×10^{-1} $(\pm 0.7 \times 10^{-2})$		0.23×10^{-1} $(\pm 0.6 \times 10^{-1})$
σ^2_e	0.14 $(\pm 0.5 \times 10^{-2})$	0.12 $(\pm 0.9 \times 10^{-2})$	0.13 $(\pm 0.5 \times 10^{-2})$	0.13 $(\pm 0.9 \times 10^{-2})$	0.98×10^{-1} $(\pm 0.2 \times 10^{-1})$	0.13 $(\pm 0.6 \times 10^{-1})$
σ^2_p	0.15 $(\pm 0.3 \times 10^{-2})$	0.15 $(\pm 0.3 \times 10^{-2})$	0.16 $(\pm 0.2 \times 10^{-2})$	0.16 $(\pm 0.2 \times 10^{-2})$	0.16 $(\pm 0.2 \times 10^{-2})$	0.16 $(\pm 0.2 \times 10^{-2})$
h^2_{men}	0.10 $(\pm 0.3 \times 10^{-1})$	0.85×10^{-1} $(\pm 0.3 \times 10^{-1})$	0.00 (± 0.0)	0.00 (± 0.0)		
h^2_{imp}					0.38 (± 0.1)	0.17×10^{-1} (± 0.7)
c^2_{men}		0.15 $(\pm 0.6 \times 10^{-1})$		0.49×10^{-1} $(\pm 0.7 \times 10^{-1})$		
c^2_{imp}					0.00 (± 0.0)	
m^2_{men}			0.16 $(\pm 0.3 \times 10^{-1})$	0.14 $(\pm 0.4 \times 10^{-1})$		
m^2_{imp}						0.15 (± 0.4)
P value		0.20×10^{-1} [a]	0.10×10^{-1} [a]	0.21 [b]<br>0.53 [c]	0.26 [b]	1.00 [c]
L	8,405.72	8,408.29	8,408.88	8,409.08	8,409.64	8,408.70
Thr	$g + e$	$g + c + e$	$g + m + e$	$g + c + m + e$	$g_s + g_d + c + e$	$g_s + g_d + m + e$
σ^2_g	0.43×10^{-1} $(\pm 0.3 \times 10^{-1})$	0.43×10^{-1} $(\pm 0.3 \times 10^{-1})$	0.11×10^{-1} (± 0.2)	0.11×10^{-1} (± 0.2)		
σ^2_{gs}					nc	nc
σ_{gsd}					nc	nc

σ^2_{gd}									nc	nc
σ^2_a	0.86×10^{-1}	$(\pm 0.5 \times 10^{-1})$	0.86×10^{-1}	$(\pm 0.5 \times 10^{-1})$	0.22×10^{-1}	(± 0.4)	0.22×10^{-1}	(± 0.4)	nc	nc
σ^2_c			0.96×10^{-7}	(± 0.0)			0.61×10^{-8}	(± 0.0)	nc	
σ^2_m					0.66×10^{-1}	(± 0.4)	0.66×10^{-1}	(± 0.4)		nc
σ^2_e	3.28		3.28		3.28		3.28		nc	nc
σ^2_p	3.33	$(\pm 0.3 \times 10^{-1})$	3.33	$(\pm 0.3 \times 10^{-1})$	3.37	(± 0.2)	3.37	(± 0.2)	nc	nc
h^2_{men}	0.26×10^{-1}	$(\pm 0.2 \times 10^{-1})$	0.26×10^{-1}	$(\pm 0.2 \times 10^{-1})$	0.65×10^{-2}	(± 0.1)	0.65×10^{-2}	(± 0.1)		
h^2_{imp}									nc	nc
c^2_{men}			0.00	(± 0.0)			0.00	(± 0.0)		
c^2_{imp}									nc	
m^2_{men}					0.20×10^{-1}	(± 0.1)	0.20×10^{-1}	(± 0.1)		
m^2_{imp}										nc
L_{ps}	–42,031.06		–42,031.06		–42,029.28		–42,029.28		nc	nc

σ^2_g = gametic variance; σ^2_{gs} = gametic variance as father; σ^2_{gd} = gametic variance as mother; σ_{gsd} = covariance; σ^2_a = additive genetic variance; σ^2_c = permanent maternal environmental variance; σ^2_m = maternal genetic variance; σ^2_e = residual variance; σ^2_p = phenotypic variance; h^2_{men} = heritability calculated with *Mendelian model*; h^2_{imp} = heritability calculated with *imprinting model*; c^2 = relative maternal environmental variance; m^2 = relative maternal genetic variance; L = logarithm of restricted estimated maximum likelihood (REML); L_{ps} = logarithm of pseudo likelihood generated according to pseudo likelihood approach by Gilmour *et al*. (2009); indices *a*, *b* and *c* indicate which models were compared in a REML log-likelihood ratio test generating *P* values; nc = not converged.

Supplementary Table S3. Overview of incremental Wald *F* values (*F*), number of numerator degrees of freedom (*DF*), number of denominator degrees of freedom (DF_{de}), and *P* values (*P*) for all fixed effects on type 1 diabetes (T1D) and rheumatoid arthritis (RA), which were sex, birth year, social economic index (SEI), number of offspring (no. offspring), medical region, SEI of the mother (SEI_{mother}), years under observation ($years_{obs}$), and whether an individual was a single child or not (single child). Threshold mixed models were used containing a gametic effect (*g*), a maternal environmental effect (*c*), a maternal genetic effect (*m*), and a residual effect (*e*)

			Mendelian model 1			*Mendelian model 2*						*Mendelian model 3*		
			g + *e*			*g* + *c* + *e*			*g* + *m* + *e*			*g* + *c* + *m* + *e*		
	effect	*DF*	DF_{de}	*F*	*P*	DF_{de}	*F*	*P*	DF_{de}	*F*	*P*	DF_{de}	*F*	*P*
T1D	birth year	67	70,942.0	131.25	0.00	70,942.0	129.88	0.00	70,942.0	130.50	0.00	70,942.0	130.50	0.00
	SEI_{mother}	5	45,953.2	10.12	1.06×10^{-9}	44,959.6	10.18	9.18×10^{-10}	45,881.1	10.09	1.13×10^{-9}	45,881.1	10.09	1.13×10^{-9}
	sex	1	42,025.9	85.08	2.99×10^{-20}	43,250.3	84.93	3.23×10^{-20}	43,993.2	86.77	1.27×10^{-20}	43,993.3	86.77	1.27×10^{-20}
	sex*birth year	67	70,942.0	2.58	2.79×10^{-11}	70,942.0	2.57	3.45×10^{-11}	70,942.0	2.57	3.45×10^{-11}	70,942.0	2.57	3.45×10^{-11}
	medical region	25	36,002.2	23.00	1.60×10^{-104}	42,239.2	22.93	2.71×10^{-104}	38,407.6	22.90	4.60×10^{-104}	38,407.6	22.90	4.60×10^{-104}
	$years_{obs}$	3	54,246.2	548.91	0.00	55,254.7	547.24	0.00	57,358.6	545.84	0.00	57,358.6	545.84	0.00
RA	birth year	56	20,869.0	4.30	1.38×10^{-24}	20,869.0	4.30	1.38×10^{-24}	20,869.0	4.27	2.63×10^{-24}	20,869.0	4.27	2.64×10^{-24}
	SEI	5	18,889.4	2.95	0.12×10^{-1}	18,889.4	2.95	0.12×10^{-1}	18,699.7	2.96	0.11×10^{-1}	18,699.7	2.96	0.11×10^{-1}
	SEI_{mother}	5	16,659.6	2.67	0.20×10^{-1}	16,659.6	2.67	0.20×10^{-1}	15,935.8	2.67	0.20×10^{-1}	15,936.0	2.67	0.20×10^{-1}
	sex	1	15,536.3	462.28	4.53×10^{-101}	15,536.3	462.28	4.53×10^{-101}	15,106.6	461.70	6.61×10^{-101}	15,106.7	461.70	6.61×10^{-101}
	sex*birth year	56	20,869.0	0.53	1.00	20,869.0	0.53	1.00	20,869.0	0.53	1.00	20,869.0	0.53	1.00
	sex*SEI	5	17,571.5	7.18	1.01×10^{-6}	17,571.5	7.18	1.01×10^{-6}	17,451.4	7.18	1.01×10^{-6}	17,451.3	7.18	1.01×10^{-6}
	sex*no. offspring	11	20,869.0	0.97	0.47	20,869.0	0.97	0.47	20,869.0	0.96	0.48	20,869.0	0.96	0.48
	no. offspring	11	18,171.0	52.54	5.72×10^{-115}	18,171.0	52.54	5.72×10^{-115}	18,007.4	52.53	6.27×10^{-115}	18,007.4	52.53	6.27×10^{-115}
	medical region	25	15,557.7	1.73	0.13×10^{-1}	15,557.7	1.73	0.13×10^{-1}	15,554.7	1.73	0.13×10^{-1}	15,554.4	1.73	0.13×10^{-1}

single child	1	16,858.3	5.97	0.15×10^{-1}	16,858.3	5.97	0.15×10^{-1}	16,618.7	5.98	0.14×10^{-1}	16,618.7	5.98	0.15×10^{-1}
$years_{obs}$	3	18,295.6	14.08	3.65×10^{-9}	18,296.1	14.08	3.65×10^{-9}	18,121.3	14.07	3.71×10^{-9}	18,124.2	14.07	3.71×10^{-9}
